# Supplementary material for: FAP-CAR-T cells reduce dystrophic muscle fibrosis, improving adeno-associated virus gene transfer efficacy
Source: Mol Ther Methods Clin Dev. 2025 Jul 30;33(3):101545. doi: 10.1016/j.omtm.2025.101545 (PMC12396263; doi:10.1016/j.omtm.2025.101545)
Supplement: Document S1. Figures S1–S6, and Tables S1 and S2 [file mmc1.pdf]

**OMTM, Volume *33***

## Supplemental information

# FAP-CAR-T cells reduce dystrophic muscle fibrosis, improving adeno-associated virus gene transfer efficacy

**Maxime Ferrand, Céline J. Rocca, Guillaume Corre, Valentina Buffa, Sophie Frin, Francine Garnache-Ottou, Elodie Bôle-Richard, Sonia Albini, Isabelle Richard, and Anne Galy**

**Figure S1**

**A**

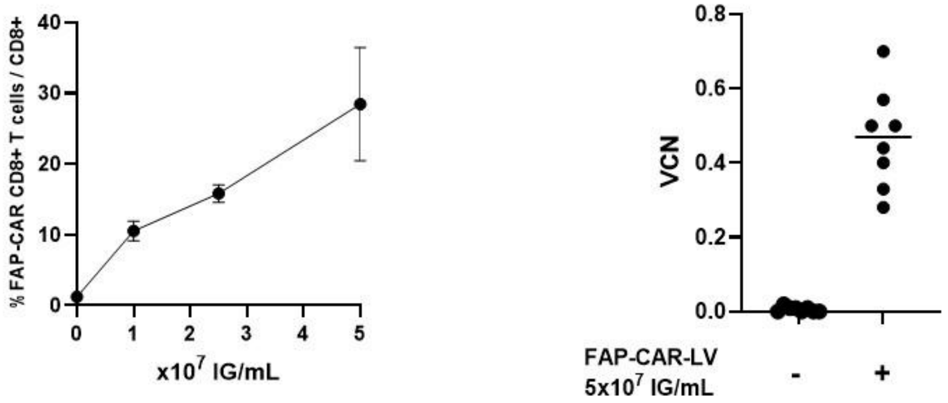


**B**

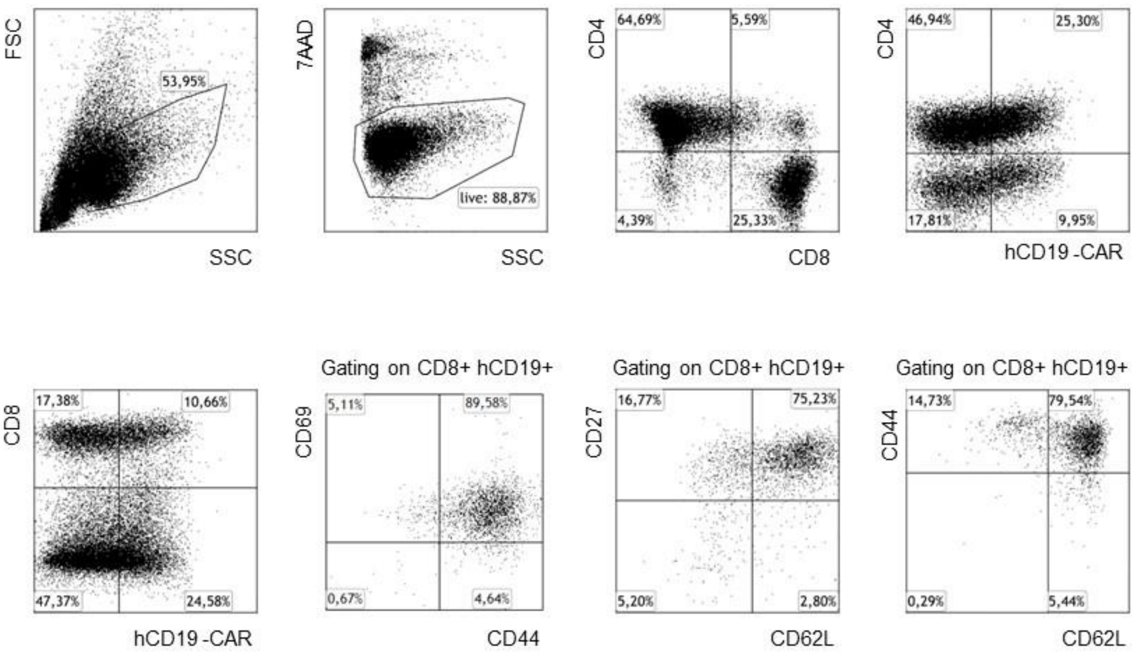


**Figure S1 legend: A. Dose effect of the FAP-CAR LV on CD8 T cells transduction.** The FAP-CAR LV was tested at 3 concentrations: 1, 2.5 and $5x10^7$ IG/mL on $1x10^6$ T cells. The left panel shows the percentage of FAP+CD8+ T cells among CD8 T cells measured by flow cytometry after 5 days of transduction, n=3 experiments. The right panel shows the vector copy number per cell (VCN) measured by ddPCR on cells transduced with $5x10^7$ IG/mL of FAP-CAR LV, n=8 experiments. **B. Representative dot plots of n=3 different flow cytometry experiments**. Plots show the gating strategy used on spleen CD3+ T cells after 7 days of culture (5 days post transduction). The percentages of live CD8+ and CD4+ cells expressing the CAR with truncated CD19 and among the CD8+ CAR+ cell population, the percentage of activated T cells expressing CD44, CD69, CD27 and CD62L are represented.

**Figure S2**

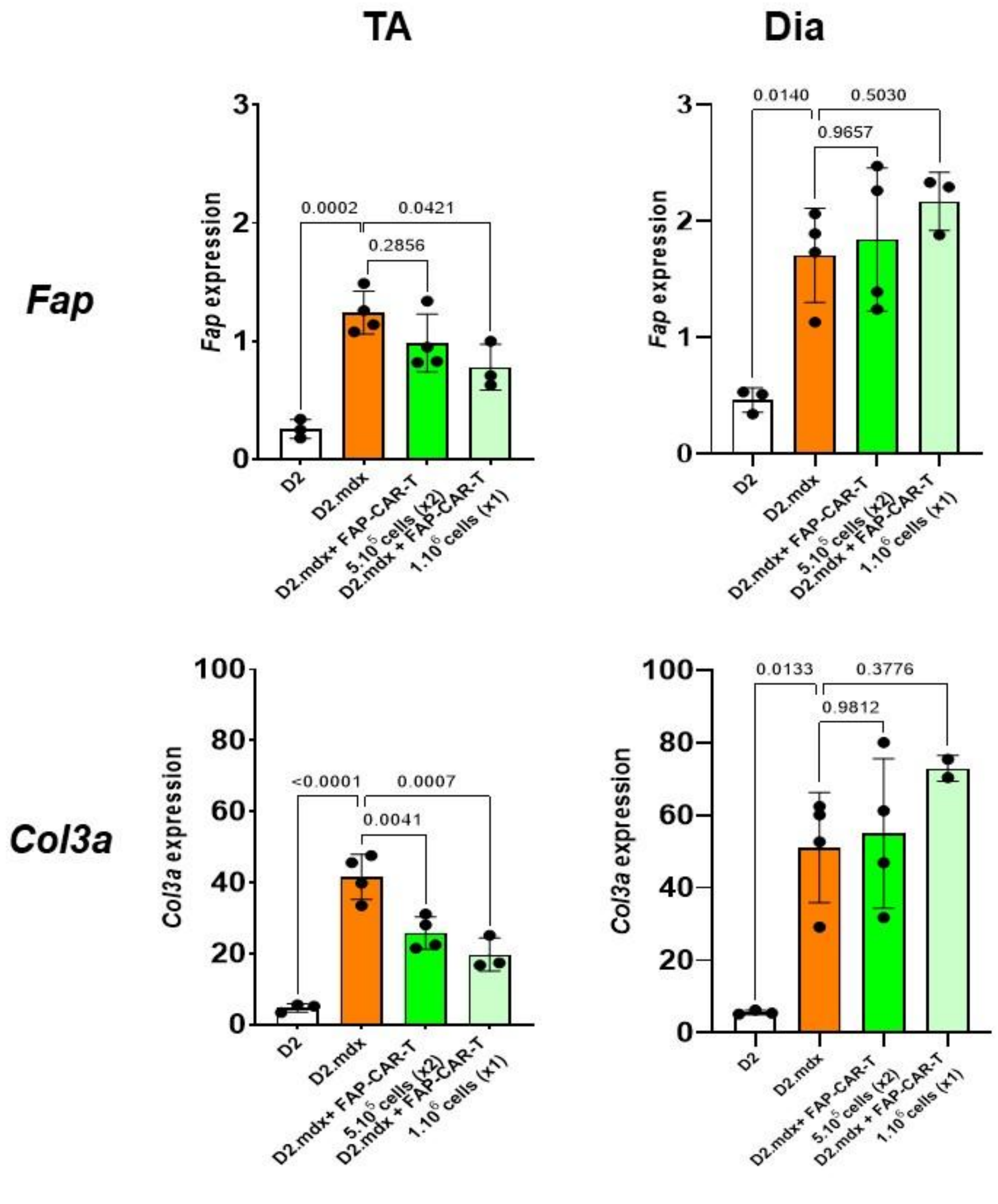


**Figure S2 legend: Gene expression after treatment:** Graphs show the mRNA expression of the genes *Fap* (top panel) and *Col3a* (bottom panel) in the muscles TA (left panel) or Dia (right panel) of either: D2 mice (white bars) or D2.mdx mice treated with control T cells (orange bars) or D2.mdx mice treated with two doses of $5x10^5$ FAP-CAR-T cells one week apart (dark green bars) or D2.mdx mice treated with one single dose of $1x10^6$ FAP-CAR-T(light green bars). Data represent values from n=3 to 4 mice per group and are relative expressions of the indicated genes normalized to *PO*. Statistical analysis used a one-way ANOVA test.

## Figure S3

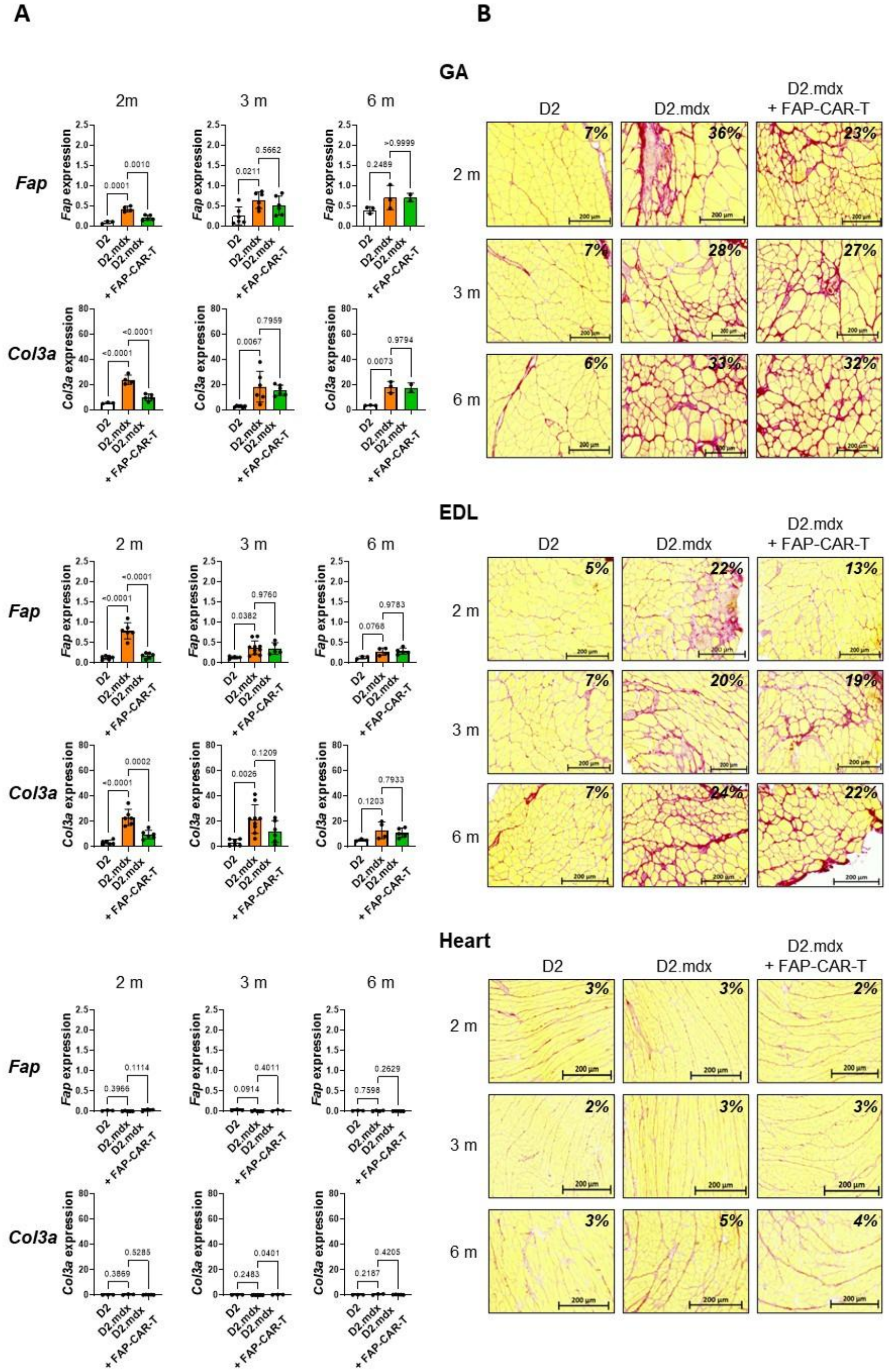

**Figure S3 legend: Gene expression (A) and histological findings (B) in different muscles after CAR-T cell treatments in mice of different age. A.** mRNA expression of the genes *Fap* (top graphs) and *Col3a* (bottom graphs) in the muscles GA (top panel), *EDL* (middle panel) and heart (bottom panel) from D2 mice (white bars) or D2.mdx mice treated with control T cells (orange bars) or with FAP-CAR-T cells (green bars) at different ages of life (n=3 to 10 mice per group). Values are relative expression normalized to *PO*. Statistical

analysis: One-way ANOVA statistic test. **B.** Representative images of Sirius red histological sections of GA, heart and EDL from mice treated as in (A). Indicated values represents the percentage of area occupied by collagen deposits relative to the total area of muscle section. The red intensity staining reveals collagen deposits (Objective: 10X).

**Figure S4**

A

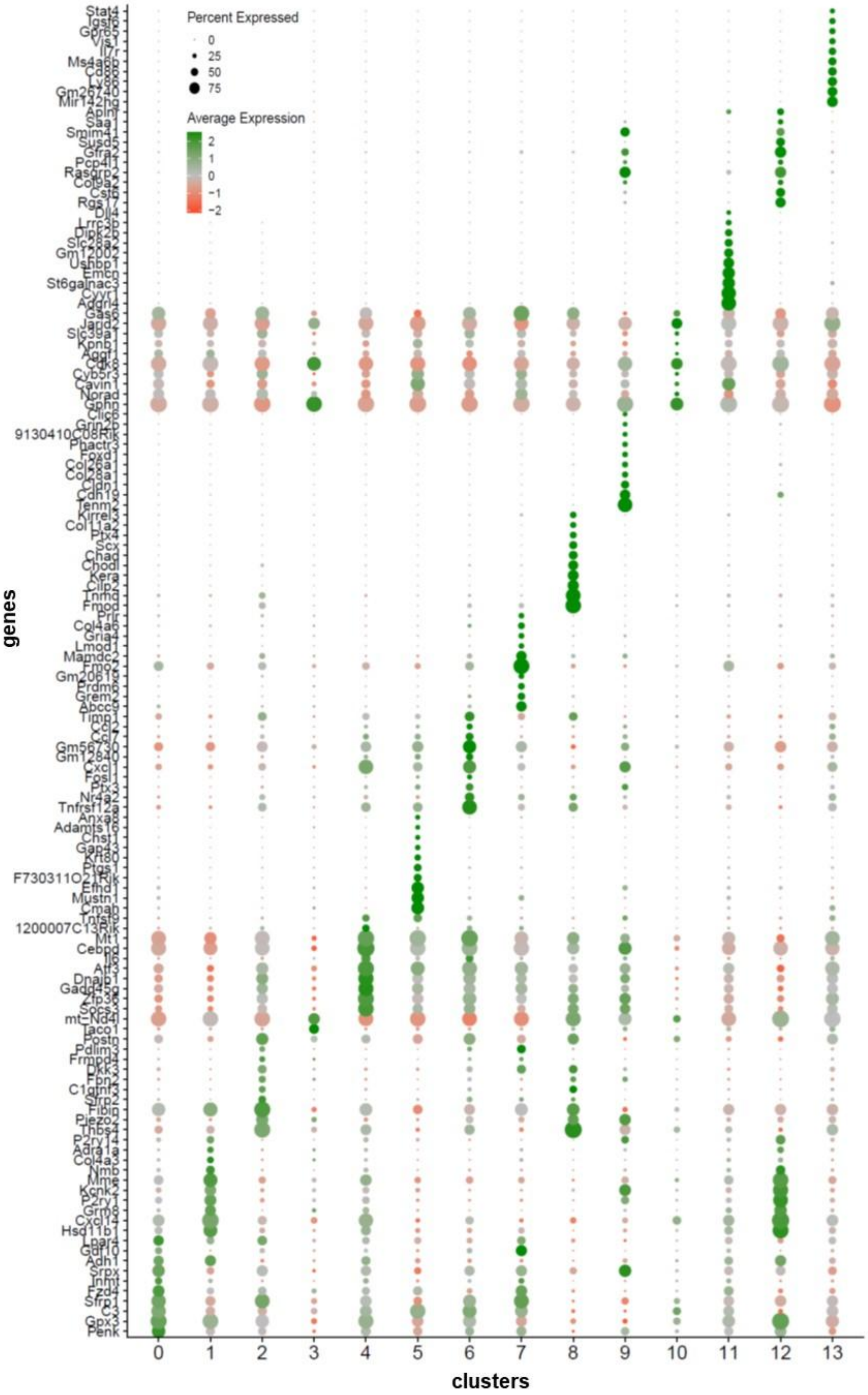


**Figure S4 legend: Dot plot Seurat in different categories of cells, based on single cell RNA seq data.** The row-scaled expression of top 10 or top 20 expressed genes is shown. The size of each dot represents the percentage of cells within the indicated cluster that express the gene. Larger dots indicate that a higher proportion of cells in the cluster express the gene, while smaller dots indicate a lower proportion. The

color intensity of each dot reflects the average expression level of the gene within the cluster. Greener colors represent higher average expression levels, while reder colors

**A. Top 10 expressed genes on *Pdgfra* sub-clusters (i.e clusters 0 to 13).**

**Figure S4 (continued)**

**B**

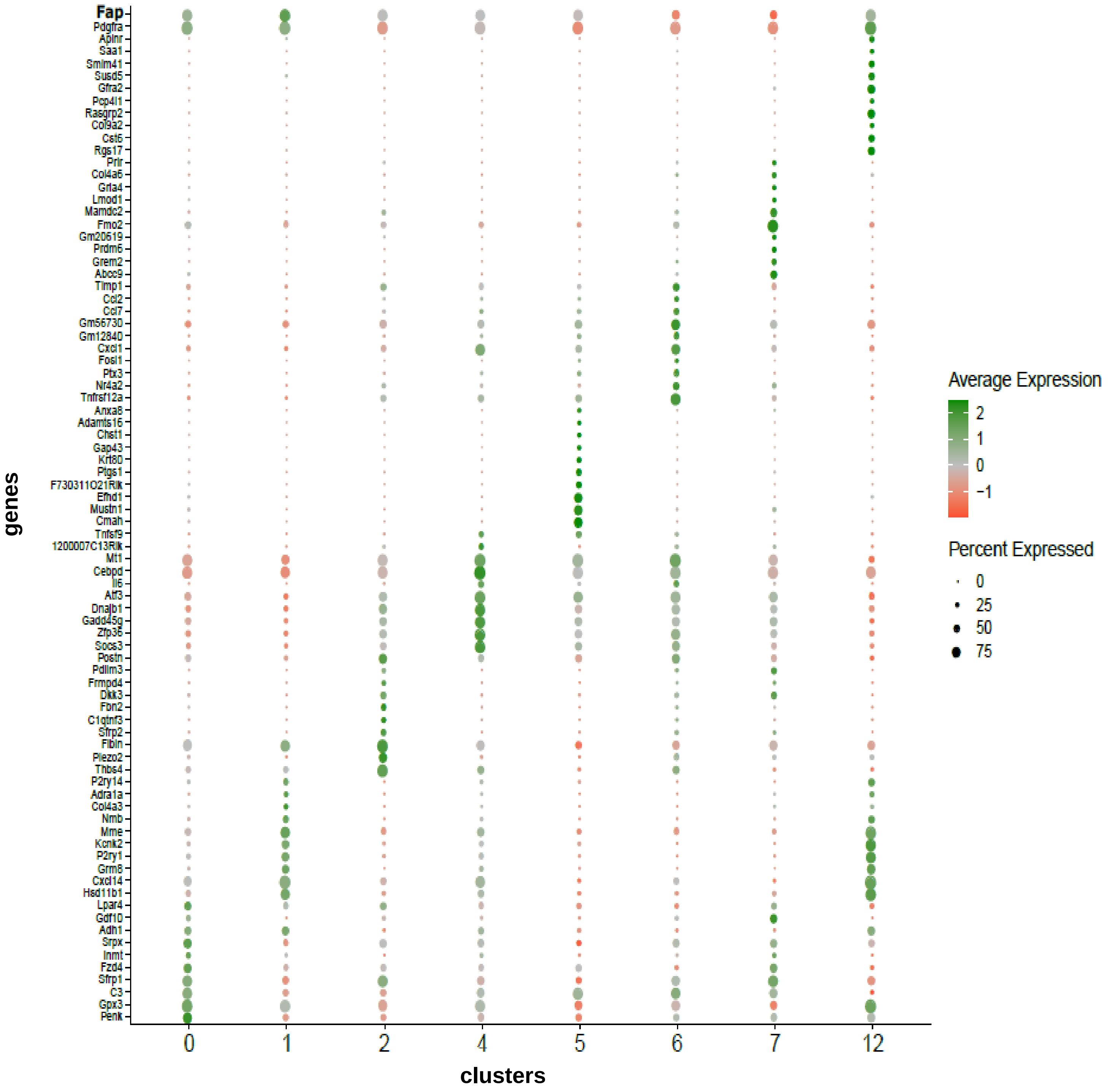


**B. Top 10 expressed genes on FAP cells sub-clusters (i.e clusters 0,1,2,4,5,6,7 and 12).**

**Figure S4 (continued)**

C

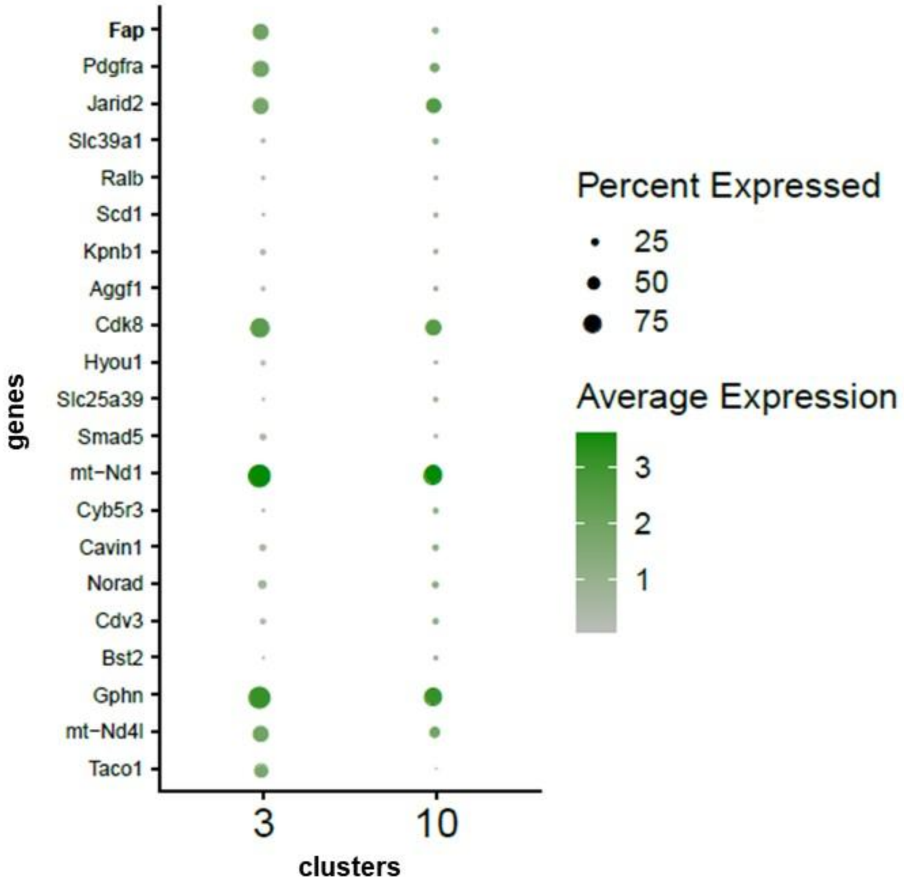


**C. Top 20 expressed genes on fibroblasts sub-clusters (i.e clusters 3 and 10).**

**Figure S4 (continued)**

**D**

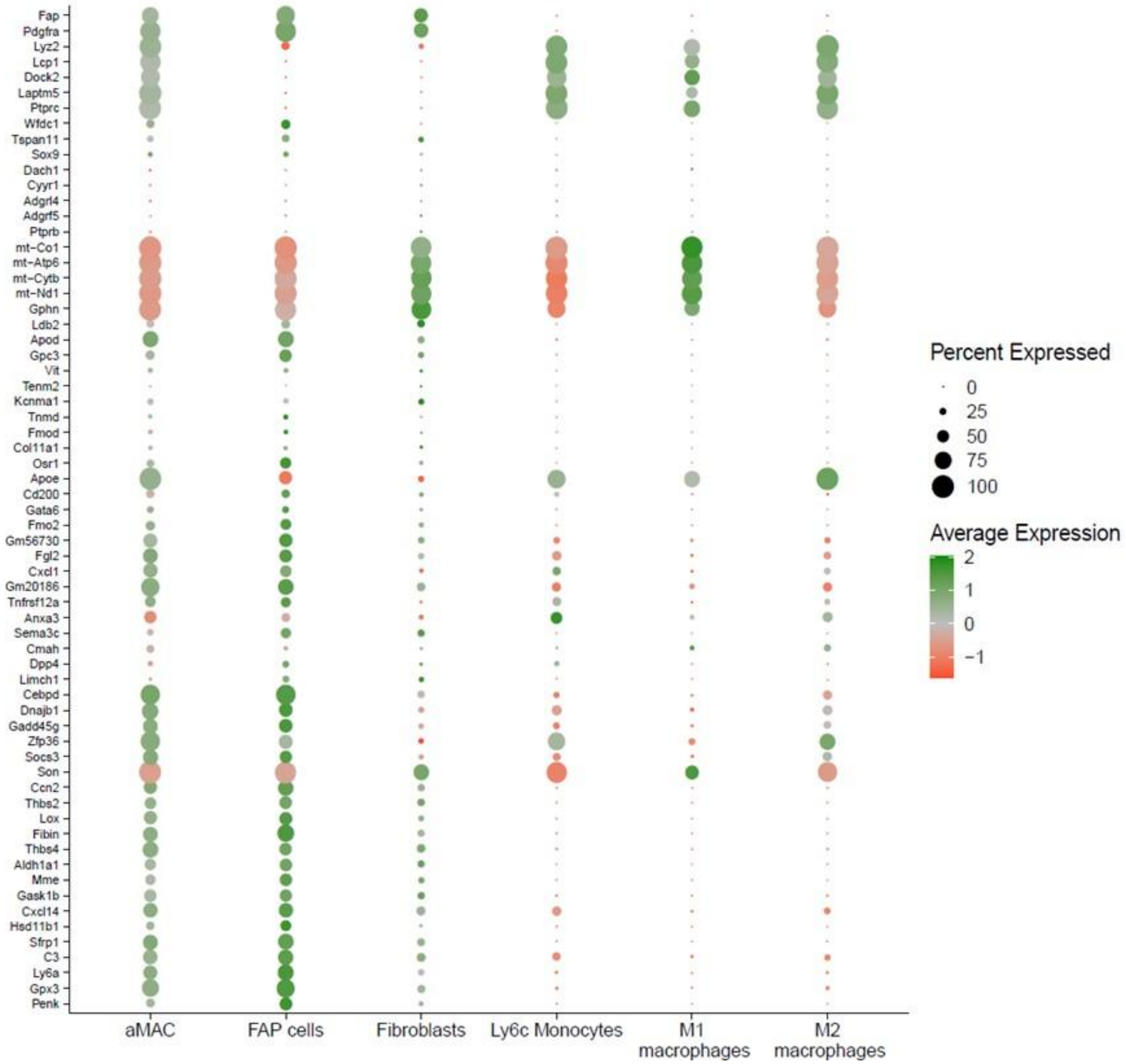


**D. Top 20 expressed genes on atypical macrophages (aMAC), FAP cells, Fibroblasts, Ly6c monocytes, M1 macrophages and M2 macrophages clusters.**

**Figure S5**

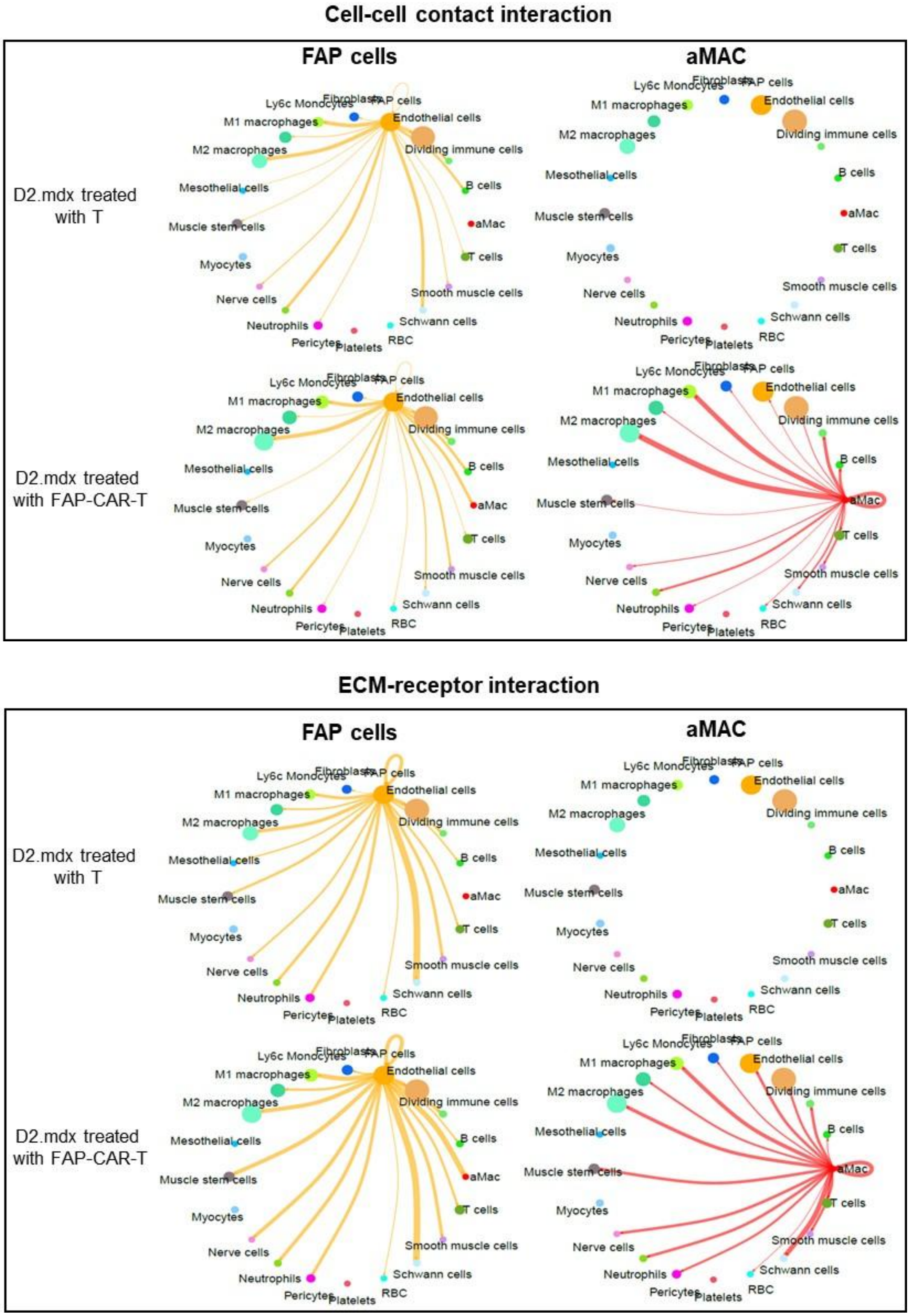


**Figure S5 legend: CellChat analysis of cellular interactions as cell-cell contact (top panels) or cell receptor-extracellular matrix (ECM) interactions (bottom panels) from single cell transcriptomic data.**

Circle plot showing the intercellular communication strength between major cell types for FAP cells (left panel) and aMAC cells (right panel) in D2.mdx mice treated with control T cells or D2.mdx mice treated with FAP-CAR-T cells. The lines indicate ligand receptor interactions between the different cell types. The thickness of the line is proportional to the probability of interaction between two cell types based on the number, the weight and the strength of ligand-receptor interactions, with loops representing autocrine circuits.

**Figure S6**

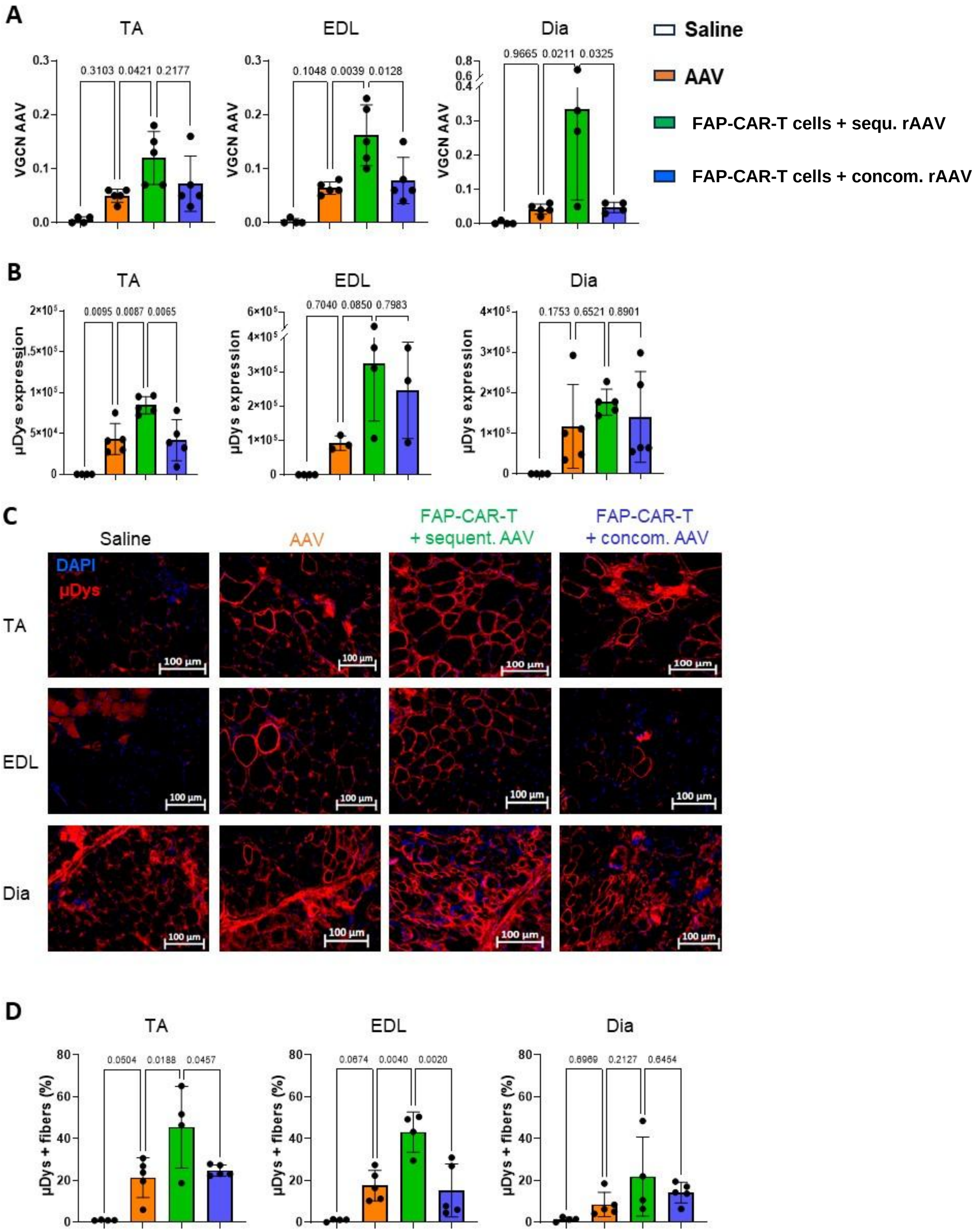


**Figure S6 legend: Temporal effects of CAR-T cell administration in combination with gene therapy.** D2.mdx mice were injected with two consecutive doses ($1x10^6$) of FAP-CAR-T cells and with 1 dose ($5.10^{12}$

vg/kg) of rAAV9-microdystrophin vector given sequentially (2 weeks after the last dose of CAR-T cells) or concomitantly (with the second dose of CAR-T cells). Control mice received only rAAV (orange bars) or saline (open bars). **A.** Quantification of vector genome viral copy number (VGCN) with ddPCR in TA, EDL and Dia in D2.mdx mice. **B.** Quantification of microdystrophin (μDys) expression after capillary western with DYS-B antibody in TA, EDL and Dia at 6 weeks post-injection in mice. Values are represented as normalized area under the curve (AUC). **C.** Representative immunohistology of microdystrophin (μDys) in the TA, EDL and Dia at 6 weeks post-injection**. D.** Quantification of dystrophin positive myofibers in TA, EDL and Dia at 6 weeks post-injection in D2.mdx mice. The percentage of dystrophin+ fibers is represented of number of dystrophin positive fibers over fibers positive for laminin. For all the panels: *N* = 3-5 mice. For panel (**A**–**F**): One-way ANOVA statistic test.

**Table S1**: **Oligonucleotide sequences**

| Oligo | Sequence 5' –> 3' |
|---|---|
| Albumin.fw | GCTGTCATCTCTTGTGGGCTGT |
| Albumin.rv | ACTCATGGGAGCTGCTGGTTC |
| Albumin.pr | CGCACGGCAAGAGGCGAGG |
| Provirus.fw | CACTCCCAACGAAGACAAGA |
| Provirus.rv | TCTGGTTTCCCTTTCGCTTT |
| Provirus.pr | TCTCTAGCAGTGGCGCCCGAACAGG |
| mTitin.fw | AAAACGAGCAGTGACGTGAGC |
| mTitin.rv | TTCAGTCATGCTGCTAGCGC |
| mTitin.pr | TGCACGGAAGCGTCTCGTCTCAGTC |
| ITRG, AAV22mers.fw | CTCCATCACTAGGGGTTCCTTG |
| ITRG, AAV18mers.rv | GTAGATAAGTAGCATGGC |
| ITRG, AAV_MGB.pr | TAGTTAATGATTAACCC |
| Microdystrophin.fw | GGTTGTGCTGGTCCAGGGCGT |
| Microdystrophin.rv | CCAACAAAGTGCCCTACTACATC |
| Microdystrophin.pr | CCGAGCTGTACCAGAGCCTGGCC |
| mPO MH181P0.fw | CTCCAAGCAGATGCAGCAGA |
| mPO M267PO.rv | ATAGCCTTGCGCATCATGGT |
| mPO M225PO.pr | CCGTGGTGCTGATGGGCAAGAA |
| MPZ (PO) ddPCR Gene Expression Assay | ID: dMmuCPE5103243, Biorad |
| FAP ddPCR Gene Expression Assay | ID: dMmuCPE5093936, Biorad |
| Col3a ddPCR Gene Expression Assay | ID: dMmuCPE5110694, Biorad |

**Table S2**: **List of antibodies used**

| Specificity | Clone / Reference | Conjugate | Provider | Dilution |
|---|---|---|---|---|
| mouse CD3 | 17A2 | Fitc | BD Pharmingen | 1 :100 |
| mouse CD4 | RM4.5 | Pacific blue | BD Pharmingen | 1 :200 |
| mouse CD8a | 53-6.7 | PE-Cy7 | Invitrogen | 1 :200 |
| mouse CD69 | H1.2F3 | Fitc | BD Pharmingen | 1 :150 |
| mouse CD44 | IM7 | PE | BD Pharmingen | 1 :100 |
| mouse CD62-L | MEL-14 | APC | BD Pharmingen | 1 :100 |
| human CD19 | HIB19 | Alexa 700 | BD Pharmingen | 1 :10 |
| human CD19 | J3-119 | APC | Beckman Coulter | 1 :10 |
| mouse CD27 | LG.3A10 | BV510 | BD Horizon | 1 :100 |
| mouse CD107a | 1D4B | PE | BD Pharmingen | 1 :20 |
| mouse laminin | polyclonal | unconjugated | Dako | 1 :1000 |
| human dystrophin | 34C5 | unconjugated | Leica Biosystem | 1 :100 |
| mouse CD11b | M1/70 | unconjugated | BD Pharmingen | 1 :40 |
| mouse FAP | polyclonal | unconjugated | Invitrogen | 1 :200 |
| goat anti-rat | polyclonal secondary | Alexa Fluor 488 | Invitrogen | 1 :600 |
| donkey anti-mouse | polyclonal secondary | Alexa Fluor 594 | Invitrogen | 1 :1000 |
