# Supplementary material for: FAP-CAR-T cells reduce dystrophic muscle fibrosis, improving adeno-associated virus gene transfer efficacy
Source: Mol Ther Methods Clin Dev. 2025 Jul 30;33(3):101545. doi: 10.1016/j.omtm.2025.101545 (PMC12396263; doi:10.1016/j.omtm.2025.101545)
Supplement: Document S2. Article plus supplemental information [file mmc2.pdf]

Molecular Therapy
Methods & Clinical Development
Original Article

FAP-CAR-T cells reduce dystrophic muscle fibrosis, improving adeno-associated virus gene transfer efficacy

Maxime Ferrand,[1,2,5] Céline J. Rocca,[1,2,5] Guillaume Corre,[1,2] Valentina Buffa,[1,2] Sophie Frin,[1,2] Francine Garnache-Ottou,[3] Elodie Bôle-Richard,[4] Sonia Albini,[1,2] Isabelle Richard,[1,2,6,7] and Anne Galy[1,2,6,7,8]

[1]Université Paris-Saclay, Univ Evry, Inserm, Integrare Research Unit UMR_S951, Genethon, 91000 Evry-Courcouronnes, France; [2]Genethon, 91000 Evry-Courcouronnes, France; [3]Université Marie et Louis Pasteur, EFS, Inserm, UMR1098 RIGHT, 25000 Besançon, France; [4]FC'innov, Bionoveo, 25000 Besançon, France

Tissue fibrosis is a pathological feature of many diseases including muscular dystrophies such as Duchenne muscular dystrophy (DMD). Fibrosis may limit the effectiveness of gene therapy in muscle impacting on viral dosing but direct evidence is lacking. Strategies to reduce skeletal muscle fibrosis are limited. The fibrosis *Fap* gene is over-expressed in the skeletal muscles of a severe mouse model of DMD, suggesting that cells expressing membrane fibroblast activation protein (FAP) could be targeted by chimeric antigen receptor (CAR)-T cells. Two consecutive administrations of FAP-specific CAR-T cells in the severe DMD model reduced collagen deposits and fibrotic biomarkers and also reduced the number of FAP-positive cells in muscle. Single cell transcriptomics revealed that FAP-CAR-T cells triggered cellular interactions with otherwise inactive muscle resident macrophages and depleted specific subsets of FAP-highly-expressing fibro-adipogenic progenitor cells, pointing to their importance in the fibrosis process. Reducing fibrosis with FAP-CAR-T cells enhanced adeno-associated virus (AAV) microdystrophin gene transfer in the model by increasing vector copies, demonstrating that fibrosis is a restriction factor for AAV gene delivery in skeletal muscle. These results provide novel insights into therapeutic strategies for DMD or other fibrotic diseases.

INTRODUCTION

Skeletal muscle fibrosis is a common hallmark of chronic progressive skeletal muscle degenerative disorders, most prominently associated with aging or with muscular dystrophies of genetic origin such as Duchenne muscular dystrophy (DMD).[1] DMD is an X-linked progressive muscular dystrophy caused by mutations in the dystrophin gene. In muscle, fibrosis manifests by the replacement of myofibers with fibroblasts and extracellular matrix (ECM) components, reducing muscle contractility and regeneration potential and function. In addition to worsening the general health of individuals, fibrosis may broadly impede the efficacy of gene therapy approaches in muscle.[2] In the fibrotic liver, hepatocyte gene delivery with adeno-associated virus (AAV) vectors or lentiviral vectors (LVs) is reduced.[3] The effects of muscle fibrosis on AAV gene transfer have not yet been directly assessed.

Fibroblast activation protein (FAP), also known as seprase, is a homodimeric cell surface glycoprotein belonging to the serine protease family. FAP was initially identified as a target for epithelial cancer diagnostic and treatment being highly expressed in stromal fibroblasts of the majority of primary and metastatic epithelial tumors while being absent from normal adult tissues.[4] Chimeric antigen receptor (CAR)-T cells specific for murine FAP were shown to deplete FAP-expressing stromal cells, reducing tumor growth in mice.[5] More recently, FAP-specific CAR-T cells were shown to reduce cardiac fibrosis in phenylephrin/angiotensin II-induced model of cardiac fibrosis.[6,7] A CAR-T cell approach for reducing fibrosis in a skeletal muscle disease has never been reported before. The levels of FAP expression in various muscles during the progression of muscular dystrophies such as DMD have not been reported in detail. Whether or not FAP-specific CAR-T cells may function in DMD is unpredictable considering the large territory of the target tissue, and because DMD is associated with significant perturbations of the immune system such as local inflammatory infiltrates in muscle tissues as well as the presence of transforming growth factor β (TGF-β) and of regulatory T lymphocytes (TLs) which may hamper immunotherapeutic approaches.[8,9]

This prompted us to test the effects of FAP-specific CAR T cell in a severe mouse model of DMD[10] and to evaluate if the reduction of

Received 18 April 2025; accepted 25 July 2025;
https://doi.org/10.1016/j.omtm.2025.101545.

[5]These authors contributed equally

[6]These authors contributed equally

[7]Senior author

[8]Present address: ART-TG, Inserm US35, 91100 Corbeil-Essonnes, France

Correspondence: Isabelle Richard, Université Paris-Saclay, univ Evry, Inserm, Integrare Research Unit UMR_S951, Genethon, 91000 Evry-Courcouronnes, France.
E-mail: richard@genethon.fr
Correspondence: Anne Galy, Université Paris-Saclay, univ Evry, Inserm, Integrare Research Unit UMR_S951, Genethon, 91000 Evry-Courcouronnes, France.
E-mail: anne.galy@inserm.fr

© 2025 The Authors. Published by Elsevier Inc. on behalf of The American Society of Gene and Cell Therapy.
This is an open access article under the CC BY license (http://creativecommons.org/licenses/by/4.0/).

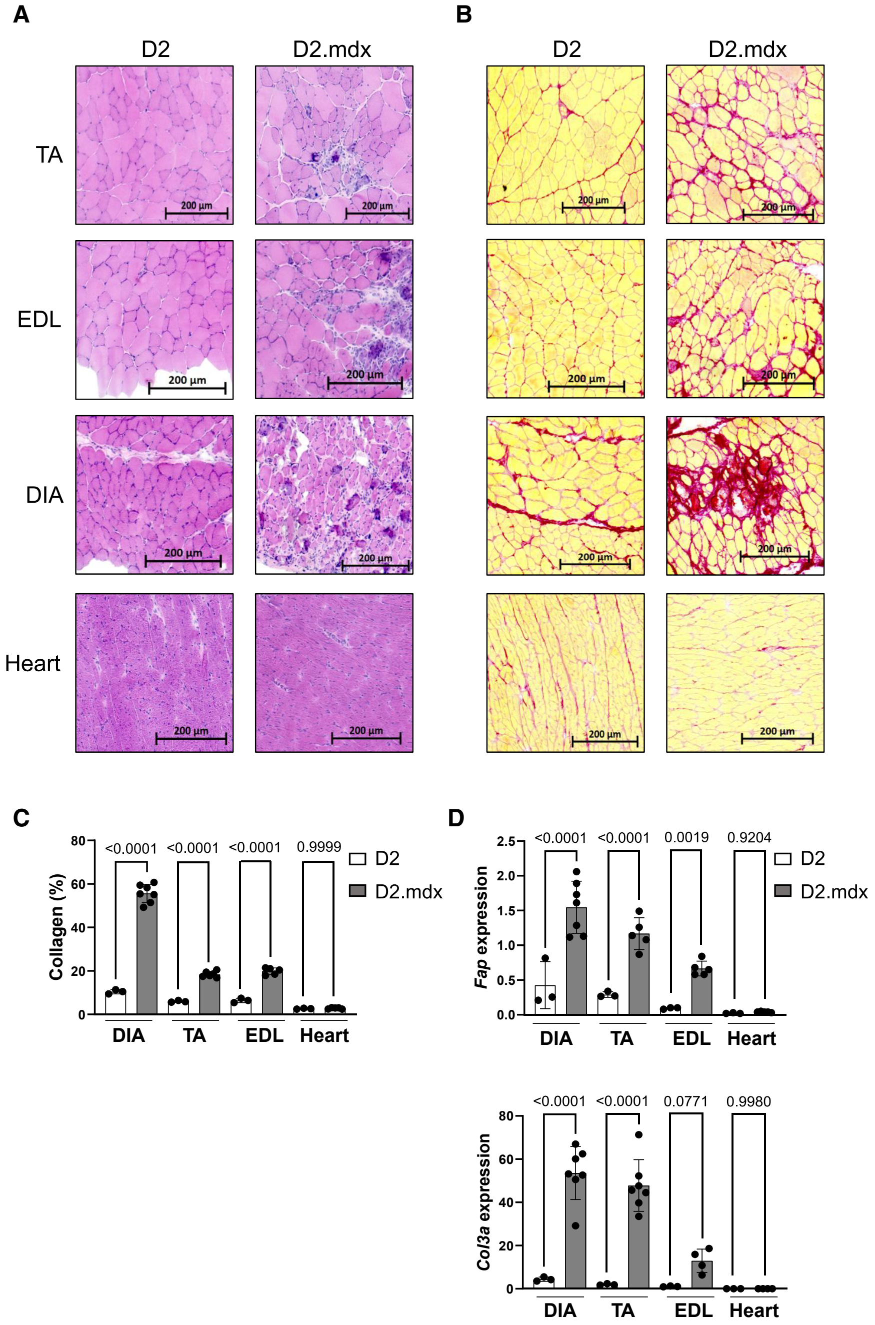

Figure 1. Fibrosis analysis in D2.mdx mice

(A) Hematoxylin and eosin staining in 3-month-old tibialis anterior (TA), extensor digitorum longus (EDL), diaphragm (DIA), and heart of D2 and D2.mdx mice. Representative images of 2 independent experiments (n = 6 to 7 independent animals). (B) Sirius red staining in TA, EDL, DIA, and heart from D2 and D2.mdx mice, showing collagen fibers in red. Representative images of 2 independent experiments (n = 6–7 independent animals). A–B Image size is shown by a scale bar. (C) Quantification of collagen deposits in DIA, TA, EDL, and heart from D2 and D2.mdx mice (n = 3 for D2 mice and n = 4 to 7 for D2.mdx mice). (D) Quantification of *Fap* (top) and *Col3a* (bottom) mRNA expression relative to the P0 gene in the TA and DIA from D2 and D2.mdx mice (n = 3 for D2 mice and n = 4 to 7 for D2.mdx mice). C–D indicate the ANOVA p values above the compared graphs, and error bars represent the standard deviation.

fibrosis by this approach could improve the efficacy of gene therapy using AAV-mediated transfer of microdystrophin.

RESULTS

Among the existing murine models of DMD, we selected for our study D2.mdx mice bearing the mdx dystrophin mutation on the DBA2/J background carrying the *LTBP4* genetic modifier increasing the phenotype severity.[11] This model exhibits severe histopathological features including fibrosis and calcifications in the diaphragm (DIA), as well as progressive collagen accumulation in limb muscles (Figures 1A–1C). Gene expression from digital droplet PCR (ddPCR) and RNA sequencing (RNA-seq) analyses revealed that limb muscles and DIA of D2.mdx mice expressed higher levels of fibrotic genes including murine *Fap* and *Col3a* compared to age-matched parental DBA2 mice (D2 mice) whereas little expression was found in the heart of D2.mdx mice or, as expected, in the muscles of D2 control mice (Figure 1D). Such differential expression results suggested that the cell surface FAP protein could represent a possible target of fibrosis for CAR T cells in D2.mdx mice, although variable effects might be observed depending on the levels of FAP in the different muscles.

FAP-specific CAR-T cells were generated by transducing pre-activated spleen D2 T cells with a self-inactivated LV. The CAR construct encodes a murine FAP-specific single chain Fv (ScFv) antibody generated from clone 73.3[12] inserted into a third-generation CAR backbone containing CD28 and 41-BB co-stimulatory domains and previously used to treat plasmacytoid leukemia in mice[13] (Figure 2A). The initial FAP-CAR construct also expressed a truncated CD19 tag, which was used to measure the CAR expression and this CD19 tag was removed in subsequent experiments to increase the vector titer (data not shown). The transduction of CD3 T cells from spleen of D2 mice cells generated an average vector-copy number (VCN) between 0.4 and 0.5 in the cell culture (Figure S1A). It produced about 95% of $CD3^+$ T cells in 7 days of culture, with about 30% of $CD8^+$ T cells expressing the CAR construct as well as CD62L, CD69,

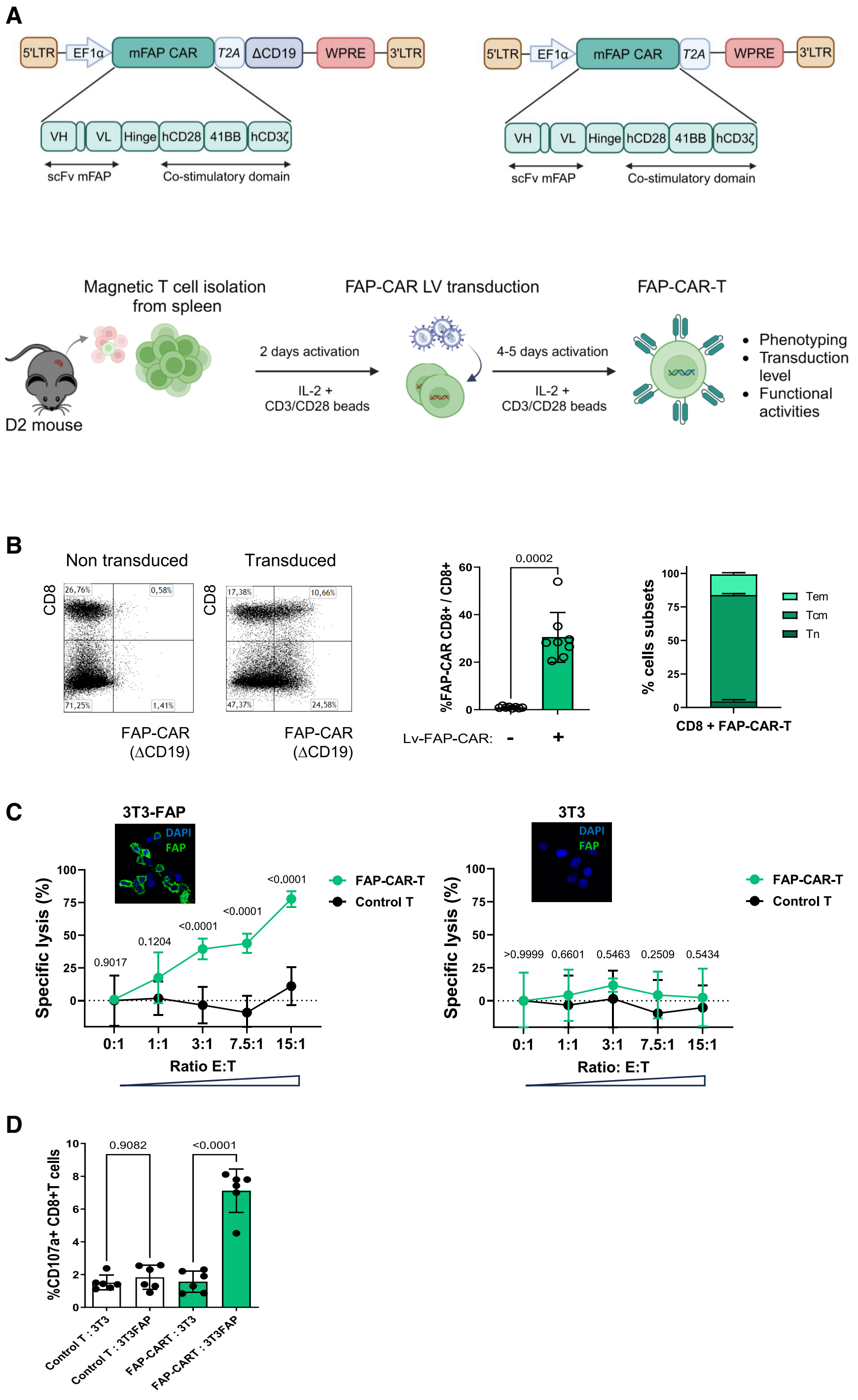

Figure 2. Generation of FAP-CAR-T cells and functional activity

(A) Schematic representation of the process used to generate FAP-CAR-T cells from D2 spleen T cells using a third-generation lentiviral vector pCCL-EF1a-scFvFAP-CD28-4.1BB-CD3ζ-T2A-ΔCD19-WPRE or pCCL-EF1a-scFvFAP-CD28-4.1BB-CD3ζ-WPRE in which the scFv against mFAP is fused to T cell signaling domains CD28, 4.1BB, and CD3ζ. (B) Flow cytometry assessment in cells cultured for 7 days. CD19 tag was used to identify CAR^{+} cells, and expressions of CD44, CD27, and CD62L to identify CD8 T cell subsets identified as Tn (naive T cells CD62L^{++}, CD44^{-}), Tcm (central memory T cells, CD62L^{++}, CD44^{++}), Tem (effector memory T cells, CD62L^{-} CD44^{++}). *p* values were calculated with Student's t test and are indicated above the compared conditions with error bars representing standard deviations (as in C–D). (C) Cytotoxic effects of cultured CAR-T cells (3 separate experiments) on 3T3-Luc2 cells expressing or not FAP. *p* values were calculated with two-way ANOVA multiple comparison statistics test. (D) Cytotoxic activity of FAP-CAR T cells was also measured by the detection of CD107a lysosomal-associated membrane protein on the surface of recently degranulated cells after coculture with 3T3-Luc2 cells FAP or not (2 experiments done in triplicate). *p* values were calculated with Student's t test.

CD27, and CD44 suggesting a central memory phenotype for the majority of CD8^{+} T cells (Figures 2B and S1B). Such cultured cells were specifically cytotoxic in a dose-dependent manner, against murine FAP-expressing cells as shown by cell lysis (Figure 2C) and degranulation assays (Figure 2D) using *Fap*-transduced 3T3 cells or parental 3T3 cell controls as target cells.

The intravenous infusion in 3-month-old D2.mdx mice, of 1 × 10^{6} FAP-specific CAR-T cell cultures as a single injection or as 2 injections of 5 × 10^{5} cells each infused 1 week apart, had limited effects on *Fap* expression in the tibialis anterior (TA) or DIA muscles, although a significant reduction of *Col3a* expression was observed in the TA but not in the DIA (Figure S2). However, two consecutive injections of 1 × 10^{6} FAP-CAR-T cells, infused one week apart, significantly reduced the expression of *Fap* and *Col3a* gene levels 2 weeks after the last CAR T cell injection in the TA (Figure 3A). These 2 injections of 1 × 10^{6} FAP-CAR-T cell each, markedly improved histological features in D2.mdx limb muscles TA, gastrocnemius (GA), and extensor digitorum longus (EDL) muscles as shown by reduced Sirius red stain of collagen fiber deposits compared to controls (e.g., mice treated with polyclonal non-transduced T cells or parental DBA2 mice) (Figures 3B, 3C, and S3B). This demonstrates for the first time that FAP-specific CAR-T cells can reduce fibrosis in dystrophic limb skeletal muscle. The anti-fibrotic effects of FAP-CAR-T cells varied in different muscles and were more pronounced when treatment was administered at an early age of life. In D2.mdx mice, the fibrotic phase begins at the end of the degeneration peak, around 1.5 months

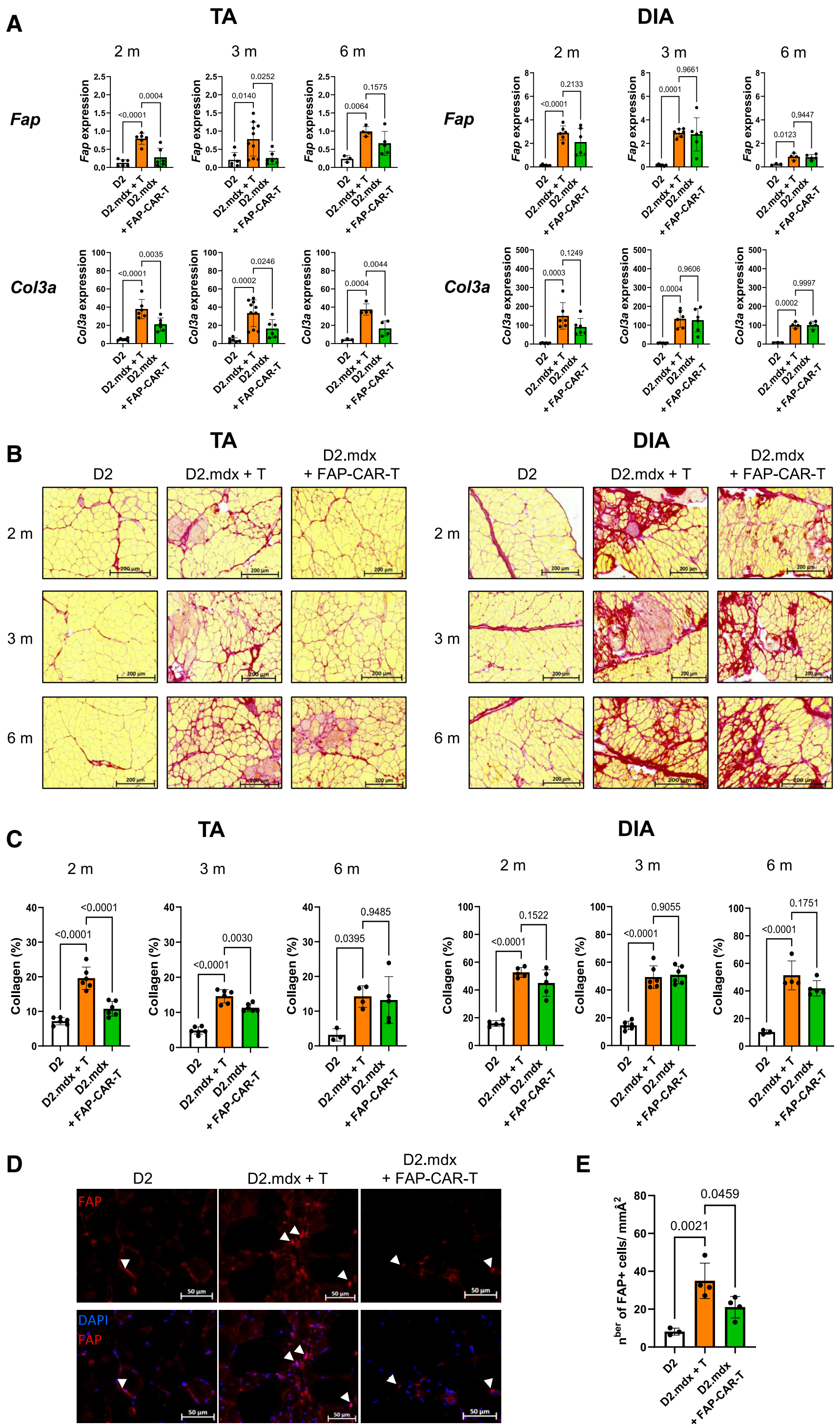

Figure 3. FAP-CAR-T cells efficiency on muscular fibrosis

(A) mRNA expression of FAP (top) and Col3a (bottom) in the TA (left) and diaphragm (DIA) (right) from D2 (white bars) and D2.mdx mice treated with polyclonal T cells (orange bars) or with FAP-CAR-T cells (green bars) (n = 3 to 9 mice per group). Statistical analysis was done by one-way ANOVA statistic test and p values are represented above the compared conditions with error bars representing standard deviations, as in C–D. (B) Representative images of Sirius red histological sections of TA and DIA, from D2 mice, D2.mdx treated with polyclonal T cells or with FAP-CAR-T cells. The red intensity staining represents collagen fibers (10× objective). (C) Quantification of the collagen deposits in the TA and DIA of mice over the entire cross-sectional area of the muscle section. The results are shown as the average percentage of fibrotic positive regions over the total area of 2 sections per muscle. Each data point represents an individual mouse. (D) Histological staining of FAP^{+} cells in the TA. Image size is shown by a scale bar; (E) Quantification of FAP+ cells per milli Angström (mmA^{2}). Statistical analysis: one-way ANOVA statistic test.

of age, rapidly reaching a first plateau at approximately 3 months, and then continues to progress more slowly until 8–9 months of age, when it reaches its maximum. In 2-month-old mice, fibrosis was reduced by FAP-CAR-T cells in the TA, GA, and EDL whereas no effect was seen in the DIA or heart (where no fibrosis was observed at that time) (Figures 3A–3C and S3). Between 3 and 6 months of age, FAP-CAR-T cells only reduced fibrosis in the TA with a marginal effect at 6 months. These kinetic results suggest that the fibrosis process cannot be reverted by the cytolytic effects of FAP-CAR-T cells once installed.

Immunostaining of TA muscle sections confirmed that FAP-CAR-T cells eliminated FAP-expressing cells present in this muscle (Figures 3D and 3E). To complete the analysis, we performed a single-cell RNA sequencing (scRNA-seq) on TA muscles five days after the last FAP-CAR-T cell injection (Figures 4A and 4B). After performing quality controls with Cell Ranger, more than 8,000 cells per condition with 1,817 gene transcripts per cell and 19,719 mean reads per cell were obtained on average. A dimensional reduction clustering analysis of these muscle mononuclear cells identified 20 cell subsets (Table 1; Figure 4A). Each cell subset was identified according to canonical gene expression profiles as described in published data.[14–25] As expected from the dystrophic process ongoing in D2.mdx mice, and as reported in mdx mice,[25] fibro-adipogenic progenitor cells (FAP cells), fibroblasts, and immune cells including several populations of macrophages were increased in D2.mdx mice treated with control T cells compared to D2 mice

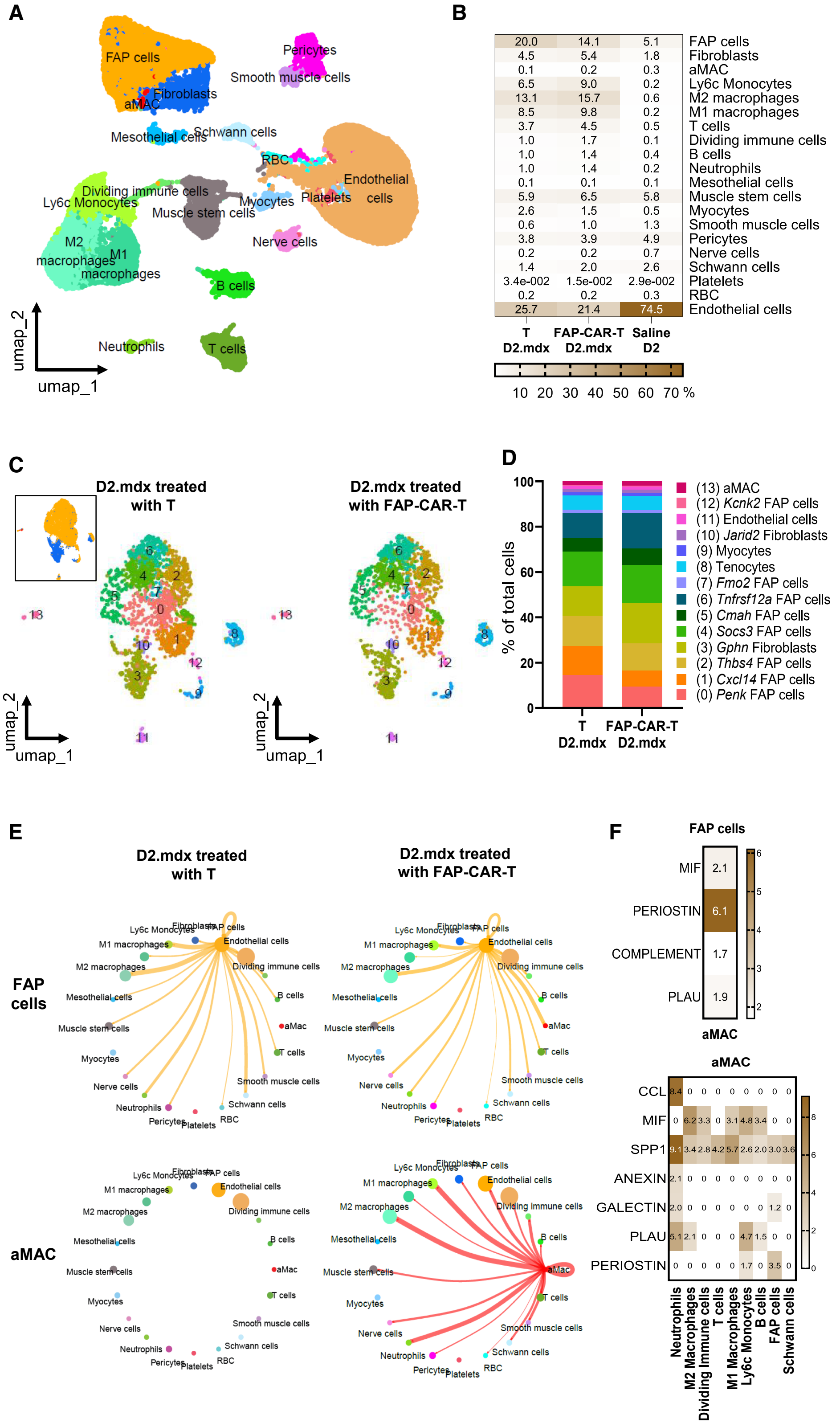

(legend on next page)

Table 1. List of major genes expressed in different cells leading to identification of clusters

Cell name	Gene symbol
Fibro-adipogenic progenitor cells (FAP cells)	*Pdgfra*, *Dcn*, *Fibn*, ***Fap***, *Colec12*, *Col6a*, *Cxcl14*, *Cd248*, *Osr1*, *CD34*
Atypical macrophages	*C1qa*, *C1qb*, *C1qc*, *Smoc2*, *Cd68*, *Cd14*, *Pdgfra*, ***Fap***
Fibroblasts	*Pdgfra*, *Dcn*, *Fibn*, *Col1a1*, *Col1a2*
Ly6c monocytes	*Ptprc*, *Lyz2*, *Cd68*, *Cd14*, *Spp1*, *Cd52*, *Ccr2*, *Tlr2*
M2 macrophages	*Ptprc*, *Lyz2*, *Cd68*, *Cd14*, *C1qc*, *C1qb*, *C1qa*
M1 macrophages	*Ptprc*, *Lyz2*, *Cd68*, *Cd14*, *Dock2*, *Pid1*, *Fcgr3*, *Csf1r*
T cells	*Ptprc*, *Itk*, *Skap1*, *Dock2*, *Cd28*, *Cd3d*
Dividing immune cells	*Mki67*, *Stmn1*, *Cd68*, *Cd74*, *Smc4*
B cells	*Ptprc*, *Cd79a*, *Cd19*, *IL21r*, *H2-Eb1*
Neutrophils	*S100a8*, *S100a9*, *Ccr1*, *Hcar2*, *Il1b*, *clec4e*
Mesothelial cells	*Gpm6a*, *Efemp1*, *Upk3b*, *Sox6*, *Wt1*, *Lrrn4*
Muscle stem cells	*Pax7*, *Myf5*, *Vcam1*, *Sytl2*, *Fgfr4*, *Chodl*
Myocytes	*Ckm*, *Myl1*, *Tnnc2*, *Tnni2*, *Tnnt3*, *Mylpf*
Smooth muscle cells	*Myh11*, *Acta2*, *Tagln*, *Synpo2*, *Ctnna3*, *Prkg1*, *Myl9*
Pericytes	*Rgs5*, *Abcc9*, *Kncj8*, *Trpc3*, *Prkg1*, *Gucy1a2*
Nerve cells	*Ccl21a*, *Mmrn1*, *Il7*, *Prox1*, *Pard6g*
Schwann cells	*Nkain2*, *Cdh19*, *S100b*, *Plp1*, *Dmd*, *Mbp*
Platelets	*F8*, *Ptprb*, *Dnase1l3*
Red blood cells	*Alas2*, *Hba-a2*, *Hbb-bt*, *Snca*
Endothelial cells	*Pecam1*, *Mecom*, *Dach1*, *Cdh5*, *Fabp4*, *Ablim3*

The selection of canonical genes is based on literature. The Fap gene is indicated in bold.

(Figure 4B). The cluster of FAP cells was specifically reduced in the TA muscles of mice injected with FAP-CAR-T cells compared to muscles of mice treated with control T cells (Figures 4A and 4B). The cluster of endothelial cells, which was globally reduced in D2.mdx mice compared to healthy D2 mice, was further reduced by CAR-T cells, probably indirectly as endothelial cells do not express the *Fap* gene. All immune cell populations, that were already increased in D2.mdx mice compared to healthy controls, were additionally increased after FAP-CAR-T cell injections.

To further specify the targeted cell subsets, a reclustering was performed on *Pdgfra* expressing cells based on uniform manifold approximation and projection (UMAP) projections showing a large cluster including fibroblasts and FAP cells as well as a small population of atypical macrophages (aMACs) sharing markers of resident macrophages as well as FAP cells (Table 1; Figure 4C). Within *Pdgfra*$^+$ cells, the analysis of distinct expression patterns of various cellular marker genes resulted in the identification of 14 clusters (Figures S4A–S4D). This analysis segregated 8 subpopulations of FAP cells (clusters 0,1,2,4,5,6,7, 12, respectively) identified by the most highly expressed genes: *Penk*$^+$, *Cxcl14*$^+$, *Thbs4*$^+$, *Socs3*$^+$, *Cmah*$^+$, *Tnfrsf12a*$^+$, *Fmo2*$^+$, and *Kenk2*$^+$ FAP cells (Figures S4A and S4B). Two types of fibroblasts were identified (clusters 3 and 10), characterized by common *Gphn*, *Cdk8*, or *Jarid2* expression but different levels of *Fap*, *Pdgfra*, and *taco-1* (Figure S4C). The small population of aMACs that was observed initially remains as a unique cluster (cluster 13) and expresses markers of hematopoietic origin such as *Ptprc* (CD45), *Mir142hg*, *Stat4*, markers of macrophagic cells such as *CD86*, *C1q*, or *ApoE* as well as markers also found on FAP cells such as *Fap*, *Pdgfra*, *Smoc2*, *Col5a1*, or *Jarid2* (Figures S4A and S4D). FAP-CAR-T cell treatment reduced only the first 2 clusters (0 and 1) which correspond to FAP cells characterized by the highest expression of the *Fap* gene (Figures 4C, 4D, S4A, and S4B). These 2 clusters of cells represent 43% of the *Pdgfra*$^+$ FAP cells. This analysis confirmed that FAP-CAR-T cells eliminated cells expressing the target *Fap* gene and showed that the target cells were contained solely in the population of FAP cells.

Intercellular communication networks, through secretion, direct cell-to-cell and ECM contacts were inferred from scRNA-seq data using CellChat,[26] (Table 2) providing graphic representations of the effects of FAP-CAR-T cell treatment on the resident muscle cells (Figure 4E). FAP-CAR-T cell treatment modified the secreted signaling interactions. FAP cells engaged in communications with aMAC cells which in turn interacted with all resident muscle cell populations in stark contrast with the limited interactions that aMAC cells have with other cells in control conditions (Figure 4E). Data for direct cell-to-cell and ECM-cell contacts confirmed the existence of FAP cell-aMAC intercellular networks (Figure S5). FAP cells interacted with aMAC cells through periostin, macrophage inhibitory factor (MIF), complement and plasminogen activator

Figure 4. Single-cell RNA-seq analysis

(A) UMAP (uniform manifold approximation and projection) representation of the final dataset from pooled data from TA and diaphragms of D2 mice or D2.mdx mice treated with polyclonal T cells or with FAP-CAR-T cells. Clusters were colored by manually assigned cell type identifiers based on skeletal muscle gene expression (see Table 1). RBC, red blood cells, aMAC, atypical macrophages. (B) Heatmap of percentage of each cluster from TA of D2 mice, D2.mdx mice treated with T cells and D2.mdx mice treated with FAP-CAR-T cells. (C) UMAP representation of re-clustering of *Pdgfra* expressing cells (i.e., the annotated FAP cells, fibroblasts, and aMAC clusters) from TAs of the different groups of mice. Clusters were colored by manually assigned cell type identifiers based on skeletal muscle gene expression and FAP cell gene expression. The upper left inset of the figure shows the parentage of *Pdgfra*-expressing cell subpopulations with FAP cells, aMAC, and fibroblasts origin-clusters. (D) Stacked bar plot showing the percentage of each cluster from the TA of the different groups of mice. (E) Circle plot showing the intercellular communication strength via secreted signaling interaction between major cell types for FAP cells, aMac in D2.mdx mice treated with control T cells or with FAP-CAR-T cells. The lines originating from a cell type indicate ligands connecting to the cell type where the receptors are expressed. The thickness of the line is proportional to the number of unique ligand-receptor interactions, with loops representing autocrine circuits. (F) Heatmap showing the dominant signaling pathways between source cells and target cells. As source cells, we tested FAP cells and aMAC and as target cells we tested neutrophils, M2 macrophages, M1 macrophages, T cells, B cells, dividing immune cells, Ly6c monocytes, Schwann cells, and aMAC cells in D2.mdx mice treated with FAP-CAR-T cells. Values represent the interaction probability between different receptor-ligand pairs within the same signaling pathway. *n*= 4 mice per group.

Table 2. List of receptor-ligand genes and pathways tested with CellChat tool for the generation of circle plot cell communication

Ligands	Tgfb1, Tgfb2, Tgfb3, Bmp4, Bmp5, Bmp6, Hbegf, Fgf7, Pdgfa, Cxcl1, Vegfa, Vegfb, Igf1, Ccl5, Ccl3, Ccl9, Ccl7, Ccl8, Cxcl2, Cxcl12, Tnfsf9, Spp1, Mif, Il6, Il34, Fasl, Tnfsf12, Tnfsf14, Nampt, Angptl1, Ptn, Postn, Mdk, Ptn, Postn, C3, Lgals9, Pros1, Plau, Pros1, Sema3d, Sema3c, Sema3e, Sema3f, Anxa1, Gas6, Psap
Receptors	TGFbR1_R2, ACVR1_TGFbR, BMPR1A_ACVR2A, Ackr1, Cxcr2, Cxcr4, Egfr, Fgfr2, Pdgfra, Pdgfrb, Flt1, Kdr, Igf1r, Ccr1, Ccr2, Ccr5, Ackr3, ITGAV_ITGB3, ITGAV_ITGB5, CD74_CD44, Tnfrsf9, Cd44, CD74_CXCR4, IL6R_IL6ST, Csf1r, Fas, Tnfrsf12a, Ltbr, ITGA4_ITGB1, Tlr4, Pirb, Cdh5, Cdh11, Sdc1, Sdc2, Sdc4, Tek, Sdc3, Lrp1, Ncl, C3ar1, ITGAM_ITGB2, P4hb, Mertk, Plaur, P4hb, NRP2_PLXNA4, Fpr1, Axl, Gpr37l1, Havcr2
Pathways	TGFb, BMP, EGF, VGF, PDGF, VEGF, IGF, CCL, CXCL, MIF, IL6, CSF, LIGTH, TWEAK, CD137, SPP1, VISFATIN, NGPTL, ANGPT, MK, PTN, PERIOSTIN, COMPLEMENT, HGF, SEMA3, ANEXIN, GAS, GALECTIN, PROS, PSAP, PLAU

(PLAU) while aMAC cells communicated through secreted phosphoprotein 1 (SPP1), CC motif chemokine ligand, MIF, annexin, galectin, and PLAU (Figure 4F). Of note, SPP1 also known as osteopontin, signals to most cell types, in particular, to neutrophils. Thus, FAP-CAR-T cells directly removed specific subsets of FAP$^+$ cell subsets which are likely involved in the initiation of the fibrotic process of skeletal muscle, and this indirectly triggered the activation of macrophages and of immune cells which appear to be involved in the resolution of the fibrotic process as well as potentially in the remodeling of the tissue.

Microdystrophin gene transfer is currently one of the most promising therapeutic avenues in DMD based on using AAVs to transfer a shortened, but still functional, version of dystrophin to fit the AAV vector capacity.[27] To determine if the reduction of fibrosis by FAP-CAR-T had an impact on the efficacy of AAV-mediated microdystrophin gene transfer in muscle, these 2 treatments were combined either sequentially or concomitantly in D2.mdx mice. Two consecutive injections of FAP-CAR-T cells prior to the administration of a suboptimal dose (5×10^{12} VG (viral genome)/kg) of an AAV 9 vector encoding a human microdystrophin under the control of the C5-12 promoter[28] led to significantly higher gene transfer in limb muscles compared to control mice treated with control polyclonal T cells (Figures 5A–5G). The level of AAV vector copies was increased by about 2.5-fold (Figure 5B) and levels of dystrophin gene (Figure 5C) and protein expression were increased by 2.5- to 3-fold by the mice FAP-CAR-T cell-treatment (Figures 5D and 5E). This augmentation was confirmed by immunostaining of dystrophin on muscle sections with 2–3 times more dystrophin positive fibers in mice treated with FAP-CAR-T cells than non-treated ones (Figures 5F and 5G). Higher dystrophin expression improved dystrophic features in muscles as shown by the reduction of inflammation, collagen deposits, calcified fibers, and the decrease of CD11b$^+$ monocyte infiltrates (Figures 6A and 6B). In contrast, the concomitant treatment of AAV at the time of the second CAR-T cell injection did not produce the gene transfer enhancing effects (Figure S6) further strengthening the hypothesis that fibrosis must be reduced to permit AAV entry into the tissue. This also shows that CAR-T cells do not act through unspecific effects to enhance AAV gene transfer.

DISCUSSION

Our results provide the first proof of concept that immunotherapy with FAP-specific CAR-T cells can reduce the fibrosis in skeletal limb muscle that results from a progressive pathological process like that of DMD. In D2.mdx mice, the fibrotic phase begins at around 1.5 months of age, rapidly reaching a first plateau at approximately 3 months and then continues to progress more slowly until 8–9 months of age, when it reaches its maximum. The anti-fibrosis effects were demonstrated at the biological, molecular, and histological level in several hind muscles; the TA, GA, and EDL of D2.mdx mice. In this model, the anti-fibrosis effects correlated with the dose of FAP-specific CAR T cells administered, and were more pronounced in young mice at 2 months of age compared to older mice. The mechanism of action of FAP-specific CAR-T cells is likely to be the killing of fibrogenic cells in muscles that secondarily prevented or delayed the accumulation of extracellular protein deposits in these tissues. This is supported by the removal of specific *Fap*-expressing cell subsets following CAR-T cell administration and is in agreement with the known mode of action of CAR- T cells in general, and with the cytotoxic specificity of FAP-specific CAR-T cells. The removal of extracellular protein deposits would not be expected from T cells that are generally not phagocytic cells, explaining why FAP-specific CAR-T cells would have no effect to reduce fibrosis when it is well established in older mice.

Fibrosis is a complex process involving multiple gene pathways and parameters[29] and the effects of FAP-CAR-T cells in our model provide novel cellular insights in this process through single-cell transcriptomics analyses. Whereas FAP cells are globally known to be pro-fibrotic, our data suggest that important functional subdivisions exist within these cells. The removal of *Penk*$^+$ FAP and *Cxcl14*$^+$ FAP cells suggests that they play a powerful role in the initiation of the fibrotic process, in agreement with their gene expression signature. The endogenous opioid precursor proenkephalin-A (*Penk*) gene has been involved in the regulation of hyperproliferative keloid fibroblasts[30] and is a biomarker of renal fibrosis.[31] *Cxcl14*$^+$ FAP cells are reportedly involved in muscle regeneration and enzymatic tissue remodeling.[20] The chemokine CXCL14 is also involved in the ATF3/Cxcl14/Jak2 signaling axis in hepatic stellate cells responsible for liver fibrosis.[9] Other genes expressed by *Penk*$^+$ *Cxcl14*$^+$ FAP cells may also play important roles in fibrosis.

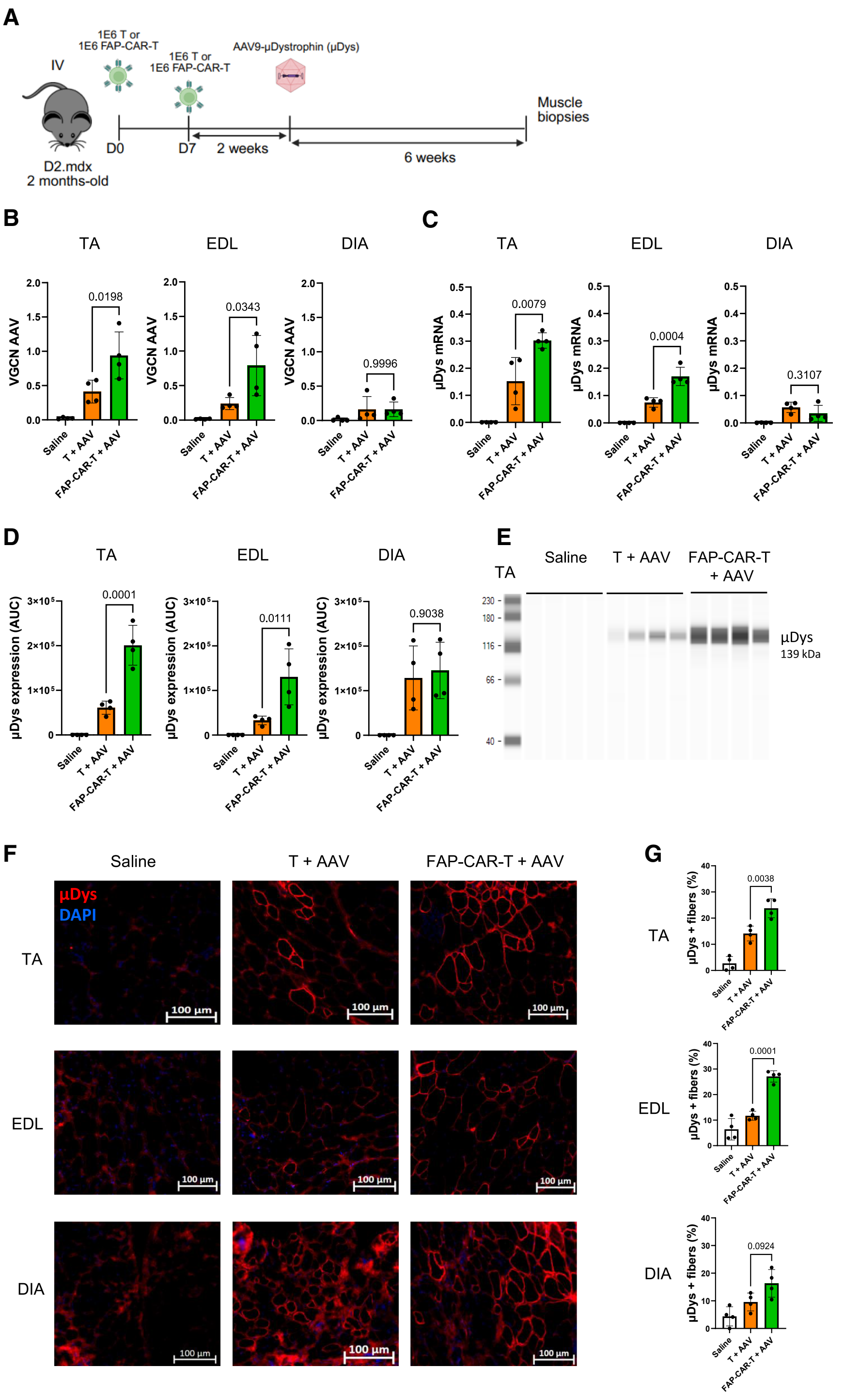

Figure 5. Combined therapy: Gene transfer efficiency

(A) Schematic representation of the *in vivo* protocol. Two-month-old D2.Mdx mice were injected or not with two consecutive doses (1×10^6) of control T cells or of FAP-CAR-T cells, and after 2 weeks of treatment the mice were injected with a single intravenous dose (5×10^{12} VG/kg) of rAAV9-microdystrophin (μDystrophin or μDys). Tissues were collected 6 weeks after the AAV injection, 4 animals were included per group and statistical analyses used one-way ANOVA tests with p values indicated above the compared conditions. (B) Quantification of VGCN with ddPCR in TA, EDL, and DIA in DBA2-MDX mice. (C) Gene expression analysis of microdystrophin in TA, EDL, and DIA of DBA2-MDX mice. Results are shown as a histogram of relative abundance of gene expression over P0 normalizer gene. (D) Quantification of microdystrophin expression after capillary western blot with DYS-B antibody in TA, EDL, and DIA at 6 weeks post-injection in mice. Values are represented as normalized area under the curve (AUC). (E) Capillary western blot of protein lysates of TA at 6 weeks post-injection in mice. Microdystrophin are visible around 139 kDa. (F) Representative microdystrophin IHF pictures of the TA, EDL, and DIA at 6 weeks post-injection in mice. Images include a scale bar. (G) Quantification of dystrophin positive myofibers in TA, EDL, and DIA at 6 weeks post-injection in D2.mdx mice. The percentage of dystrophin+ fibers is represented of number of dystrophin positive fibers over fibers positive for laminin.

Cells other than FAP cells may also play a role in the fibrosis process, including macrophages. In muscular dystrophies, M1 macrophages are chronically activated and promote muscle injury while the reparative M2 macrophages functions are perturbed and pathologically repurposed to promote fibrosis through various pathways.[32–34] Interestingly, the administration of FAP-CAR-T cells specifically triggered an atypical population of macrophages (aMAC cells) interacting with FAP cells and with other immune cells by strongly engaging the periostin and complement pathways already reported in macrophages of mdx mice.[25] Such aMAC cells expressed markers of resident tissue macrophages such as *Folr2* or *C1q* as well as low levels of FAP cell genes including *Fap* and *Pdgfra*. They interacted with all immune cells, particularly with neutrophils, through the Spp1 (osteopontin) pathway which is known to promote fibrosis in mdx mice.[35] These aMACs expressed low levels of the *Lgals3* gene and may include or resemble a recently described population of *Lgals3* expressing gal3+ macrophages found in dystrophic muscle and implicated in a temporal regulation of muscle fibrosis and regeneration through the Spp1 pathway.[25] In our study, the aMAC cells may simply respond to CAR-T cells by eliminating FAP cells or may have specific functions in the control of fibrosis in dystrophic muscle.

Altogether, our data provide cellular insights that may be useful to guide novel therapeutic strategies against fibrosis in addition to those

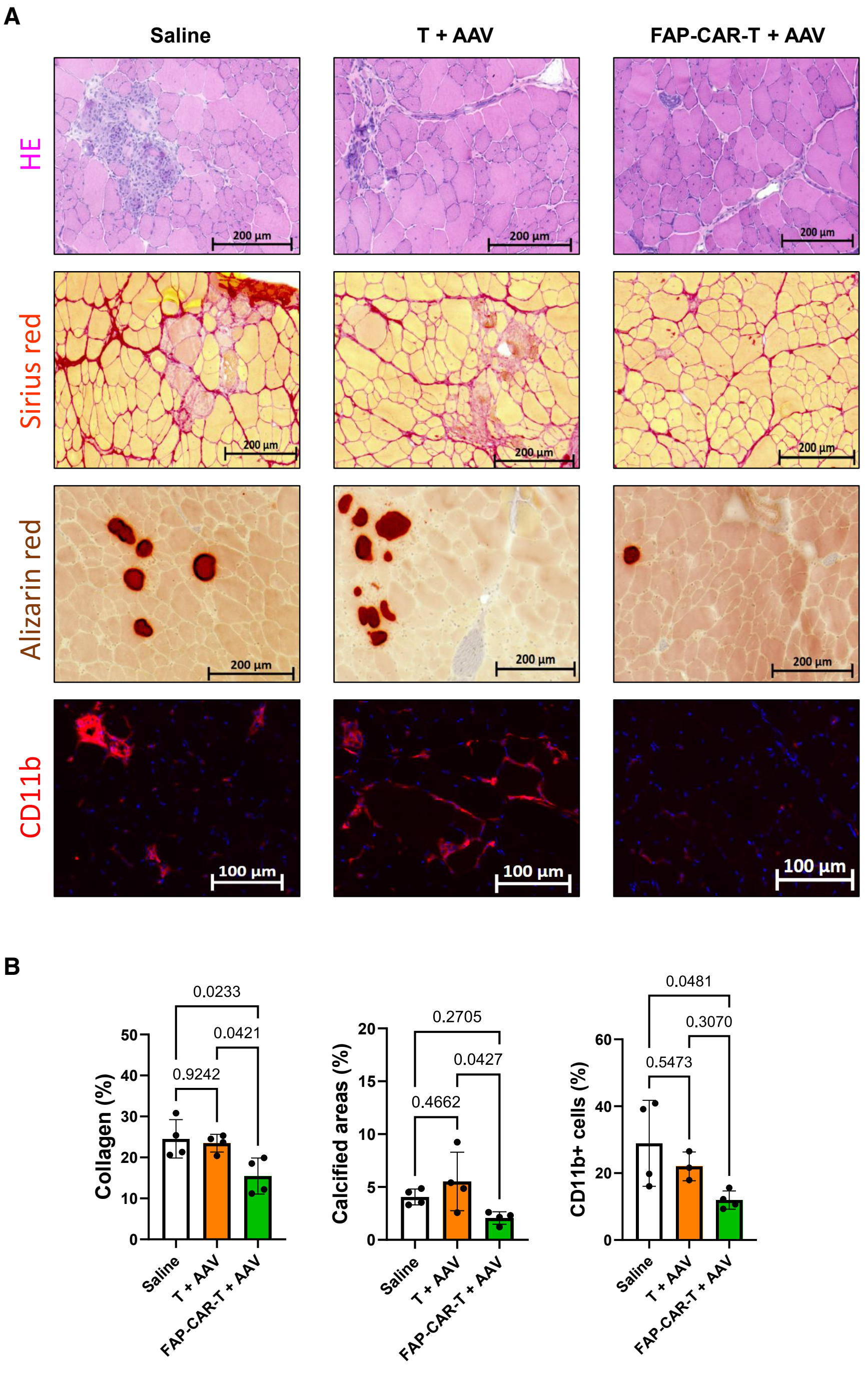

Figure 6. Combined therapy: Histology effects

Mice were treated as in Figure 5A. (A) Representative images with scale bars, of hematoxylin and eosine, Sirius red, alizarine red coloration, and CD11b staining in the TA, EDL, and diaphragm of Dba2-mdx mice 6 weeks post-injection. For Sirius red coloration, red-colored regions are highly fibrotic and contain collagen deposits. For alizarine coloration, brown areas are indicated as a region containing calcium deposits. For CD11b staining, red signals indicate monocytes cells. (B) Quantification of the collagen, calcium deposits area and CD11b cells in the TA of mice. The results are shown as the percentage of fibrotic positive or calcium positive regions over the total area of the muscle cut for colorations. The results for CD11b staining are shown as the percentage of CD11b^{+} cells after nuclei segmentation on R software. Statistical p values are indicated above the compared conditions.

currently being actively explored.[19] They suggest that anti-fibrotic immunotherapy with FAP-specific CAR-T cells should be implemented early on to remove fibrogenic cells before extracellular protein content is too important. They also suggest that specific subsets of FAP cells and of macrophages may play an important role in the fibrogenic process. Further confirmation of these findings is warranted, but is beyond the scope of this study.

The anti-fibrotic effects of FAP-CAR-T cells were mainly observed in the TA, GA, and EDL hind muscles but not in the DIA, suggesting that fibrosis in this muscle is regulated by different pathophysiological mechanisms. Very high levels of *Fap* and *Col3a* genes are already expressed in the DIAs of 2 month-old D2.mdx mice and may be difficult to reduce with CAR-T cells. It is also possible that FAP-CAR-T cells do not reach the DIA efficiently linked either to this tissue's properties (e.g., vascularization) or to the CAR T cell properties. Future modifications in the CAR-T cell administration protocol, including higher doses, repeated administration schedules or perhaps the inclusion of a lympho-depletion protocol as for human CAR-T cells, may increase the efficacy of CAR-T cells to reach a greater territory of muscles.

Microdystrophin gene transfer is currently an approved gene therapy for some DMD patients in several countries.[36] The high doses of AAV8 or AAV9 vector used pose a risk of potential occurrence of serious adverse events[37]; therefore, strategies aiming to reduce AAV dosage would be important to increase the safety of systemically administered AAV gene therapy at high doses. By reducing fibrosis prior to administration of AAV, FAP-CAR-T cell treatment can relieve a block in AAV transduction and therefore could be used to reduce the vector dose. Our data suggest that fibrosis blocks the entry of rAAV into muscle because CAR-T cells increased the delivery of vector copies in muscle having a proportional effect to increase transgene expression. Presumably, the ECM and collagen deposits create a physical barrier limiting access of the particles to the tissue, by

masking/perturbing the expression of vector attachment factors or entry receptors; or by sequestration of the particles away from myofibers. Coherent with our findings, hepatocyte gene delivery with systemically administered AAV or lentiviral vectors is also reduced in several models of liver fibrosis induced by various chemicals or by genetic mutations, although differential effects were observed depending on the models.[3] Further studies are therefore needed to better understand the mechanisms of rAAV entry in fibrotic muscle tissues.

Overall, as shown here with CAR-T cells in a model of DMD, it appears that interfering with the fibrosis initiation steps may have significant benefits in gene therapy especially when high doses of rAAV vector are needed. We were not able to demonstrate a physiological benefit on muscle strength from the use of FAP-CAR-T cells either alone or in combination with low dose rAAV coding for microdystrophin (data not shown). Such dose of vector was 10 times lower than the dose of AAV9-μDys required to achieve a beneficial effect on muscle strength because our objective was to assess a potential enhancing effect of FAP-CAR-T treatment on AAV gene delivery, an effect that would not be detectable if 100% dystrophin-positive fibers were already obtained. Further study is therefore needed to design effective therapies of DMD combining AAV and FAP-CAR-T cells. However, reducing or avoiding fibrosis is probably a useful strategy to be able to reduce the vector dose in DMD gene therapy and to increase the safety of the approach based on reduced viral dosing effects. Our findings are likely to also improve the efficacy of gene therapy of other dystrophic pathologies involving fibrosis including limb-girdle muscular dystrophies. Preventing the induction of fibrosis by removing fibrogenic cells through CAR-T cells or by other means may also improve a broad range of pathological conditions or to reduce the effects of aging mediated by fibrosis.

MATERIALS AND METHODS

Plasmid constructions

Construction of the anti-FAP CAR plasmid: The *pCCL-EF1a-scFvFAP-CD28-4.1BB-CD3ζ-T2A-ΔCD19-WPRE* plasmid was constructed by cloning the VH-VL sequences of the anti-mouse FAP antibody 73.3[12] described in WO 2014/055442 in place of the VH-VL scFvCD123 sequences of the third generation CAR reported in a study by Bole-Richard e al.[13] cloned into a *pCCL-EF1a-scFvCD123-CD28-4.1BB-CD3ζ-T2A-ΔCD19-WPRE* LV that was provided by A. Galy and F. Garnache-Ottou. A variant *pCCL-EF1a-scFvFAP-CD28-4.1BB-CD3ζ-WPRE* was constructed by excising the truncated CD19 (*ΔCD19)* tag from the initially constructed FAP-CAR lentiviral plasmid.

Construction of the FAP plasmid: The *pCCL-SFFV-mFAP-WPRE* plasmid was generated by inserting the m*FAP* cDNA (purchased from R&DSystems [RDC2905]) into a pCCL lentiviral plasmid using the SFFV retroviral promoter (kind gift from A. Thrasher, UCL London). For all plasmids, colonies were screened by digestion and correct clones were confirmed by sequencing.

Lentiviral production and generation of stably transduced target and control cell lines

LVs were produced by transient transfection of HEK293T cells using calcium phosphate and 4 plasmids including a transfer plasmid and 3 accessory plasmids (HIV-1 gagpol, HIV-Rev, and VSV-G) and titered as infectious genome (IG)/mL on HCT116 cells using the digital droplet PCR (ddPCR) provirus primers normalized to albumin as previously reported[38] (see primers and probes sequences in Table S1). The following transfer plasmids were used: pCCL-EF1a-scFvFAP-CD28-4.1BB-CD3ζ-T2A-ΔCD19-WPRE, pCCL-EF1a-scFvFAP-CD28-4.1BB-CD3ζ-WPRE, pCCL-SFFV-mFAP-WPRE, and pCCL-SFFV-Luc2-WPRE.

Stable cell lines expressing FAP, Luc2, or both transgenes, 3T3-FAP, 3T3-Luc2, and 3T3-FAP-Luc2, were generated by lentiviral transduction of the NIH-3T3 fibroblast cell line (ATCC) using the LV described previously. Transgene expression was confirmed by immunochemistry or bioluminescence assays.

FAP-CAR-T cells preparation and phenotyping

DBA2 primary murine splenic T cells were isolated using the "Pan T cell Negative Selection" kit (Miltenyi Biotec) and transduced with the indicated LV. The cells (1×10^6 cells/well/mL) were cultured in RPMI-1640 supplemented with glutamine, penicillin/streptomycin (antibiotics), 10% fetal bovine serum (FBS), 50 μM beta-mercaptoethanol, in the presence of 50 μg/mL IL-2 + CD3/CD28 activation beads at a ratio of 1:1 (Gibco) (complete medium). After 48 h, the cells were transduced with the CAR-T LV (2×10^7 IG/mL LV on 1×10^6 cells/mL for a multiplicity of infection [MOI] of 20) in the presence of the poloxamer Lentiboost (0.5 mg/mL) (Revvity) to enhance transduction. After overnight incubation, cells were expanded in complete medium for another 4 days. T cells transduction efficacy was determined either by flow cytometry using an anti-human CD19 when using the LV bearing the truncated CD19 tag or by ddPCR to determine the VCN per cell using provirus-specific ddPCR primers in relation to copies of the murine *Titin* gene (Table S2). Control polyclonal T cells were generated in parallel with the same activation process but without LV transduction.

Phenotyping of CAR T cells was done after 7 days of culture using fluorescent immunostaining with a panel of anti-mouse antibodies against CD3, CD8, CD4, CD62L, CD44, CD69 (see supplemental Table S2), and the viability marker 7AAD, using the CytoFLEX S cytometer (Beckman Coulter). Data were analyzed with the Kaluza software (Beckman Coulter).

Luciferase assay for FAP-CAR-T cell recent cytotoxic activity

3T3-Luc2 control cells and 3T3-FAP-Luc2 target cells (5,000) were plated in a 96-well plate in 100 μL of DMEM medium supplemented with glutamine, antibiotics and 10% FBS. The following day, FAP-CAR-T cells or control non-specific T cells were added at 4 different concentrations in 100 μL of medium containing 5,000, 15,000, 37,500, or 75,000 cells, to co-culture cells and targets for 24 h. At the end of the co-culture, 100 μL of supernatant medium was

removed from each well and mixed with 100 μL of luciferin solution (Bright-Glo Luciferase assay, Promega) to measure bioluminescence levels extemporaneously using a luminometer (560 nm) providing relative light units (RLU). Sixplicate wells were averaged and the percent specific lysis was calculated with the following equation: % specific lysis = 100 − (RLU-treated cells × 100)/mean RLU non-treated cells).

Degranulation assay to measure FAP-specific cytotoxicity

Control 3T3 cells and 3T3-FAP target cells (5,000) were plated in a 96-well plate in 100 μL of complete DMEM medium. The following day, 100 μL containing 75,000 FAP-CAR-T cells or control non-specific TL were added to the corresponding wells together with brefeldin A (1/2000) and 20 μL/mL of anti-CD107a. After 6 h incubation at 37°C, cells were washed with PBS-1X and stained with anti-CD8 antibody. After adding the viability marker 7AAD, cells were analyzed by flow cytometry.

Animal care and use

All animal procedures were approved by the National Ethical Committee, C2EA-51 (Evry-Courcouronnes, France), and the French Ministry of Research (MESRI) and received a national agreement number (APAFiS no. 33622 and APAFiS no. 45009). For this entire study, only males were used. The DBA2 (B6; 129S4-DBA2tm1Cpr/J, strain no. 000671) (D2) controls mice were purchased directly from Charles River Laboratories) while the DBA2/mdx (D2.B10-Dmdmdx/J, strain no. 013141) (D2.mdx) mice were bred and obtained from the Center d'Exploration et de Recherche Fonctionnelle Expérimentale (CERFE) in Evry, France. The D2. mdx strain originates from Jackson Laboratories (D2.B10-*Dmd*^{mdx}/J, strain no. 013141) and has been bred at CERFE for about 8–10 generations.

D2.mdx mice aged 2, 3, or 6 months were anesthetized and injected via the retro-orbital vein with 5×10^5 or 1×10^6 of control T cells or specific FAP-CAR-T cells resuspended in 100 μL PBS. Mice received a second injection of the same cells one week later. Two weeks after the second cell injection, D2.mdx mice and D2 age-matched control mice were euthanized to collect various muscles for molecular and histological analyses.

For rAAV treatment, D2.mdx mice received 2 consecutive injections of 1×10^6 FAP-CAR-T cells as described previously, and unless indicated otherwise, were infused 2 weeks later with 5.10^{12} VG (viral genome)/kg of rAAV9-microdystrophin vector. Control D2.mdx mice were treated similarly only with polyclonal control T cells instead of CAR-T cells. After 6 weeks, mice were euthanized to collect various muscles for molecular and histological analyses.

RNA extraction and gene expression analysis

TA, GA, EDL, DIA and heart collected from mice were directly in RNAlater for total RNA extraction using the RNeasy fibrous tissue kit (QIAGEN) following manufacturer's recommendations. RNA (1,000 ng) was then reverse transcribed using the Verso cDNA Synthesis kit (Thermo Fisher Scientific, Waltham, MA, USA). For the ddPCR, 1× of ddPCR Supermix for Probes no dUTP (Bio-Rad), 16 ng of complementary DNA and the following primers sets (Table S1) were used: ddPCR Gene Expression Assay:Fap, Mouse (Bio-Rad, 10031252), ddPCR Gene Expression Assay:Col3a1, Mouse (Bio-Rad, 10031252), and ddPCR Gene Expression Assay:Mpz, Mouse (Bio-Rad, 10031255). PCR conditions were 95°C for 10 min + 40 times (94°C for 30 s, 60°C for 1 min) + 98°C for 10 min. Droplets were generated using the droplet generator QX200 (Bio-Rad) and results analyzed using QuantaLife software (Bio-Rad).

Histological and immunostaining analyses

Muscles collected from mice were immediately snap frozen in liquid nitrogen and processed for histological microscopy. Transverse cryosections (8–10 μm) from frozen muscles were air-dried, stained with Sirius red, red alizarin, and hematoxylin and eosin and examined with an Axioscan Z1 automated slide scanner (Carl Zeiss, Oberkochen, Germany) using a Plan APO 10X/0.45 NA objective.

Immunostainings were done by incubating muscle sections overnight at 4°C with the primary antibodies against mouse laminin, dystrophin, CD11b or FAP revealed by a goat secondary antibody conjugated with Alexa Fluor 594 dye (Table S2). The sections were then mounted using DAPI (4',6-diamidino-2-phenylindole) -Fluoromount-G (Southern Biotech, Birmingham, AL, USA) and visualized on an Axioscan Z1 automated slide scanner (Zeiss) with a Plan APO 10X/0.45 NA objective. Image processing and addition of scale bars was done with the Zen Lite software (Zeiss).

Automatic image analysis

From the scanned immunostained muscle sections, the percentage of dystrophin-positive myofibers and the fluorescence marking intensity in myofibers were quantified using automatic image analysis. Myofiber cytoplasmic regions enclosed within the membrane staining (Laminin) were segmented by morphological segmentation after contrast enhancement, artifact filtering (FiJi software 2.0.0-rc/1.52p, Morpholib plugin v 1.4.1), and image size adjustment to capture the membrane regions. Nuclei were detected from the DAPI channel using the local maxima detection. The fluorescence intensity in each object (fibers, fiber membranes, and nuclei) was measured for each channel together with fibers shape and size. Nuclei were associated to their parent fiber using the R software and all fluorescence and shape data were aggregated together. Non-fiber objects were filtered-out based on shape, size, and fluorescence criteria. Positive myofibers for any channel (laminin, microdystrophin, and nuclei) were detected based on the fluorescence distribution of negative control sections to determine the percentage of microdystrophin-positive fibers.

Collagen deposits and calcified fibers on muscle section were quantified using the open-source QuPath Software. Two pixel classifiers were created to train the software on 3 muscle sections to identify representative regions (collagen region), one to delineate the tissue to be analyzed and the other to identify the regions of collagen

deposits. The surface area occupied by collagen was quantified in relation to the total muscle section surface area.

Genomic DNA extraction and viral genome copy number analysis

Genomic DNA was extracted from the muscles using a NucleoMag Pathogen kit (Macherey-Nagel, Allentown, PA, USA) according to the manufacturer's instructions and was purified using the KingFisher Flex purification system (Thermo Fisher Scientific). Droplet digital PCR was performed for the detection of viral genome copy number (VGCN) per diploid genome using AAV_ITR specific primers and to the *titin* genomic region (Table S1) for normalization.

Capillary western

Muscle proteins were extracted in radio-immunoprecipitation assay (RIPA) buffer supplemented with Protease Inhibitor Cocktail EDTA-free (Roche), quantified by the bicinchoninic acid (BCA) method (Pierce BCA protein assay kit, Invitrogen) and deposited on the Simple Western Jess system (ProteinSimple, Bio-Techne, Minneapolis, MN, USA) using a 12–230 kDa separation module. Microdystrophin detection was performed using the antibody DysB (NCL-DYSB, Leica, 1:20) and chemiluminescence detection was quantified by the Compass software.

Single-cell RNA sequencing studies

Muscle cells were isolated from the TA and DIA tissues using enzymatic dissociation (dispase II [2.4 U/mL], collagenase A [2 μg/mL], Dnase1 [10 ng/mL] in HBSS + BSA 0.2%]) followed by successive filtrations through 100, 70, and 40 μm strainers to remove aggregates. Cell viability was assessed using Trypan blue exclusion, and only samples with a viability greater than 85% were used for sequencing. A target of 20,000 cells per sample was loaded into a microfluidics-based droplet system (10× Genomics Chromium, Pleasanton, CA, USA) to generate single-cell gel beads in emulsion using 10× Genomics Chromium Single Cell 3′ kit in which reverse transcription is performed to synthesize cDNA. This step was followed by amplification, fragmentation, and ligation of Illumina sequencing adaptors. The resulting libraries were then size-selected and quantified using an Agilent Bioanalyzer or similar platform. Libraries were sequenced on an Illumina NovaSeq 6000 platform using paired-end 150 bp reads (Illumina, Inc., San Diego, CA, USA). Sequencing was performed at a depth of around 20,000 reads per cell to achieve sufficient coverage for transcriptomic analysis. Raw data was demultiplexed and aligned to the reference genome (precompiled GRCm39-2024-A, 10× Genomics) using the Cell Ranger pipeline (10× Genomics, v.8.0.1). Sequencing data was processed using Seurat (v.4.0.0) in R software. Cells were filtered based on quality control metrics including the number of genes detected per cell (cells with fewer than 200 genes or more than 2,500 genes were excluded) and the proportion of mitochondrial genes (cells with >5% mitochondrial gene expression were excluded). Additional filtering included the exclusion of cells with low UMI (unique molecular identifier) counts and potential doublets.

Samples were integrated using the harmony package (v.1.2.1) cells were clustered using the FindClusters function, which employs a graph-based clustering approach with the Louvain algorithm. Clustering resolution was set to 0.6 to optimize cluster granularity. To visualize the clusters, UMAP was performed, and cells were projected into a two-dimensional space based on the top PCs. To identify differentially expressed genes between clusters, the FindAllMarkers function in Seurat was used. Genes expressed in more than 10% of any cluster's cells were considered. Also, a minimal difference of 40% of expressing cells between each cluster and all the others was set to select interesting genes. Finally, a log2 fold change of at least 1 is used to filter-out genes with minor difference in expression. Marker genes for each cluster were annotated based on known biological functions.

Cell-cell communication analysis between ligand and receptor was performed using the CellChat package (V2.1.2) on the harmony integrated dataset.

The mouse CellChatDB was used to investigate the "secreted signaling," "ECM-receptor," and "cell-to-cell contact" categories of interaction across identified clusters.

Interactions supported by at least 10 cells are considered for plotting and analysis.[26]

Statistical analysis

All data were analyzed using GraphPad Prism 10.2.2 software. Parametric tests such as t tests and ANOVA were used for statistical comparison. To compare the two groups, a statistical comparison was performed using an unpaired t test. To compare multiple groups, we used one-way ANOVA with Tukey's correction for multiple comparison tests. Graphs were generated using Graphpad Prism v.10.2.2 or R version 3.6.2. The figures display the mean ± standard deviation.

DATA AVAILABILITY

Requests can be sent to Anne Galy PhD, currently at ART-TG, Inserm 30 rue H. Desbrères, 91100 Corbeil-Essonnes, France (anne.galy@inserm.fr) and Isabelle Richard, Genethon, Integrare research unit, 1 bis rue de l'Internationale 91001 Evry-Courcouronnes, France (richard@genethon.fr).

ACKNOWLEDGMENTS

This work was supported by funds from Genethon and from Inserm. We acknowledge the help with RNA-seq by the platform Single Cell Biomarkers UtechS and Florence Jagorel from the Biomics Platform, C2RT at Institut Pasteur (28 rue du docteur Roux, 75015 Paris, France) supported by France Génomique (ANR-10-INBS-09) and IBiSA. We are also grateful to Nathalie Bourg-Alibert and to the Bioexperimentation and Imaging platforms of Genethon for their help with breeding mice, histology, and microscopic image quantification.

AUTHOR CONTRIBUTIONS

M.F. and C.J.R. share first authorship. M.F. has contributed to methodology, investigation, validation, analysis, and writing. C.J.R. has contributed to the study conceptualization, methodology, investigation, and validation. G.C. contributed to methodology, investigation, software, and data analysis. S.F. and V.B. contributed to the investigations. F.G.-O. and E.B.-R. contributed to resources. S.A. contributed to methodology and resources. A.G. and I.R. are senior authors and co-corresponding authors, and both contributed to the study conceptualization, supervision, funding, writing, review and editing, as well as providing resources.

DECLARATION OF INTERESTS

A.G., C.J.R., M.F., S.A., and I.R. are inventors of a patent entitled "Immunotherapy of skeletal myopathies using anti-FAP CAR-T cells" published on April 11, 2024, and referenced as WO/2024/074727.

SUPPLEMENTAL INFORMATION

Supplemental information can be found online at https://doi.org/10.1016/j.omtm.2025.101545.

REFERENCES

1. Mahdy, M.A.A. (2019). Skeletal muscle fibrosis: an overview. Cell Tissue Res. *375*, 575–588. https://doi.org/10.1007/s00441-018-2955-2.
2. Cordova, G., Negroni, E., Cabello-Verrugio, C., Mouly, V., and Trollet, C. (2018). Combined Therapies for Duchenne Muscular Dystrophy to Optimize Treatment Efficacy. Front. Genet. *9*, 114. https://doi.org/10.3389/fgene.2018.00114.
3. Simoni, C., Nozi, J., Starinieri, F., La Bella, T., Manta, E., Negri, C., Biffi, M., Norata, R., Rocchi, M., Sanvito, F., et al. (2025). Liver fibrosis negatively impacts in vivo gene transfer to murine hepatocytes. Nat. Commun. *16*, 2119. https://doi.org/10.1038/s41467-025-57383-8.
4. Fitzgerald, A.A., and Weiner, L.M. (2020). The role of fibroblast activation protein in health and malignancy. Cancer Metastasis Rev. *39*, 783–803. https://doi.org/10.1007/s10555-020-09909-3.
5. Bughda, R., Dimou, P., D'Souza, R.R., and Klampatsa, A. (2021). Fibroblast Activation Protein (FAP)-Targeted CAR-T Cells: Launching an Attack on Tumor Stroma. ImmunoTargets Ther. *10*, 313–323. https://doi.org/10.2147/ITT.S291767.
6. Aghajanian, H., Kimura, T., Rurik, J.G., Hancock, A.S., Leibowitz, M.S., Li, L., Scholler, J., Monslow, J., Lo, A., Han, W., et al. (2019). Targeting cardiac fibrosis with engineered T cells. Nature *573*, 430–433. https://doi.org/10.1038/s41586-019-1546-z.
7. Rurik, J.G., Tombácz, I., Yadegari, A., Méndez Fernández, P.O., Shewale, S.V., Li, L., Kimura, T., Soliman, O.Y., Papp, T.E., Tam, Y.K., et al. (2022). CAR T cells produced in vivo to treat cardiac injury. Science *375*, 91–96. https://doi.org/10.1126/science.abm0594.
8. Juban, G., Saclier, M., Yacoub-Youssef, H., Kernou, A., Arnold, L., Boisson, C., Ben Larbi, S., Magnan, M., Cuvellier, S., Theret, M., et al. (2018). AMPK Activation Regulates LTBP4-Dependent TGF-beta1 Secretion by Pro-inflammatory Macrophages and Controls Fibrosis in Duchenne Muscular Dystrophy. Cell Rep. *25*, 2163–2176. https://doi.org/10.1016/j.celrep.2018.10.077.
9. Li, X., Lin, L., Li, Y., Zhang, W., Lang, Z., and Zheng, J. (2024). ATF3-mediated transactivation of CXCL14 in HSCs during liver fibrosis. Clin. Transl. Med. *14*, e70040. https://doi.org/10.1002/ctm2.70040.
10. Hammers, D.W., Hart, C.C., Matheny, M.K., Wright, L.A., Armellini, M., Barton, E. R., and Sweeney, H.L. (2020). The D2.mdx mouse as a preclinical model of the skeletal muscle pathology associated with Duchenne muscular dystrophy. Sci. Rep. *10*, 14070. https://doi.org/10.1038/s41598-020-70987-y.
11. Coley, W.D., Bogdanik, L., Vila, M.C., Yu, Q., Van Der Meulen, J.H., Rayavarapu, S., Novak, J.S., Nearing, M., Quinn, J.L., Saunders, A., et al. (2016). Effect of genetic background on the dystrophic phenotype in mdx mice. Hum. Mol. Genet. *25*, 130–145. https://doi.org/10.1093/hmg/ddv460.
12. Wang, L.C.S., Lo, A., Scholler, J., Sun, J., Majumdar, R.S., Kapoor, V., Antzis, M., Cotner, C.E., Johnson, L.A., Durham, A.C., et al. (2014). Targeting fibroblast activation protein in tumor stroma with chimeric antigen receptor T cells can inhibit tumor growth and augment host immunity without severe toxicity. Cancer Immunol. Res. *2*, 154–166. https://doi.org/10.1158/2326-6066.CIR-13-0027.
13. Bole-Richard, E., Fredon, M., Biichle, S., Anna, F., Certoux, J.M., Renosi, F., Tse, F., Molimard, C., Valmary-Degano, S., Jenvrin, A., et al. (2020). CD28/4-1BB CD123 CAR T cells in blastic plasmacytoid dendritic cell neoplasm. Leukemia *34*, 3228–3241. https://doi.org/10.1038/s41375-020-0777-1.
14. De Micheli, A.J., Laurilliard, E.J., Heinke, C.L., Ravichandran, H., Fraczek, P., Soueid-Baumgarten, S., De Vlaminck, I., Elemento, O., and Cosgrove, B.D. (2020). Single-Cell Analysis of the Muscle Stem Cell Hierarchy Identifies Heterotypic Communication Signals Involved in Skeletal Muscle Regeneration. Cell Rep. *30*, 3583–3595.e5. https://doi.org/10.1016/j.celrep.2020.02.067.
15. Dell'Orso, S., Juan, A.H., Ko, K.D., Naz, F., Perovanovic, J., Gutierrez-Cruz, G., Feng, X., and Sartorelli, V. (2019). Single cell analysis of adult mouse skeletal muscle stem cells in homeostatic and regenerative conditions. Development *146*, dev174177. https://doi.org/10.1242/dev.174177.
16. Giordani, L., He, G.J., Negroni, E., Sakai, H., Law, J.Y.C., Siu, M.M., Wan, R., Corneau, A., Tajbakhsh, S., Cheung, T.H., and Le Grand, F. (2019). High-Dimensional Single-Cell Cartography Reveals Novel Skeletal Muscle-Resident Cell Populations. Mol. Cell *74*, 609–621.e6. https://doi.org/10.1016/j.molcel.2019.02.026.
17. Heezen, L.G.M., Abdelaal, T., van Putten, M., Aartsma-Rus, A., Mahfouz, A., and Spitali, P. (2023). Spatial transcriptomics reveal markers of histopathological changes in Duchenne muscular dystrophy mouse models. Nat. Commun. *14*, 4909. https://doi.org/10.1038/s41467-023-40555-9.
18. Muhl, L., Genové, G., Leptidis, S., Liu, J., He, L., Mocci, G., Sun, Y., Gustafsson, S., Buyandelger, B., Chivukula, I.V., et al. (2020). Single-cell analysis uncovers fibroblast heterogeneity and criteria for fibroblast and mural cell identification and discrimination. Nat. Commun. *11*, 3953. https://doi.org/10.1038/s41467-020-17740-1.
19. Muraine, L., Bensalah, M., Butler-Browne, G., Bigot, A., Trollet, C., Mouly, V., and Negroni, E. (2023). Update on anti-fibrotic pharmacotherapies in skeletal muscle disease. Curr. Opin. Pharmacol. *68*, 102332. https://doi.org/10.1016/j.coph.2022.102332.
20. Oprescu, S.N., Yue, F., Qiu, J., Brito, L.F., and Kuang, S. (2020). Temporal Dynamics and Heterogeneity of Cell Populations during Skeletal Muscle Regeneration. iScience *23*, 100993. https://doi.org/10.1016/j.isci.2020.100993.
21. Saleh, K.K., Xi, H., Switzler, C., Skuratovsky, E., Romero, M.A., Chien, P., Gibbs, D., Gane, L., Hicks, M.R., Spencer, M.J., and Pyle, A.D. (2022). Single cell sequencing maps skeletal muscle cellular diversity as disease severity increases in dystrophic mouse models. iScience *25*, 105415. https://doi.org/10.1016/j.isci.2022.105415.
22. Santos, M.D., Gioftsidi, S., Backer, S., Machado, L., Relaix, F., Maire, P., and Mourikis, P. (2021). Extraction and sequencing of single nuclei from murine skeletal muscles. STAR Protoc. *2*, 100694. https://doi.org/10.1016/j.xpro.2021.100694.
23. Uapinyoying, P., Hogarth, M., Battacharya, S., Mázala, D.A.G., Panchapakesan, K., Bönnemann, C.G., and Jaiswal, J.K. (2023). Single-cell transcriptomic analysis of the identity and function of fibro/adipogenic progenitors in healthy and dystrophic muscle. iScience *26*, 107479. https://doi.org/10.1016/j.isci.2023.107479.
24. Wosczyna, M.N., and Rando, T.A. (2018). A Muscle Stem Cell Support Group: Coordinated Cellular Responses in Muscle Regeneration. Dev. Cell *46*, 135–143. https://doi.org/10.1016/j.devcel.2018.06.018.
25. Coulis, G., Jaime, D., Guerrero-Juarez, C., Kastenschmidt, J.M., Farahat, P.K., Nguyen, Q., Pervolarakis, N., McLinden, K., Thurlow, L., Movahedi, S., et al. (2023). Single-cell and spatial transcriptomics identify a macrophage population associated with skeletal muscle fibrosis. Sci. Adv. *9*, eadd9984. https://doi.org/10.1126/sciadv.add9984.
26. Jin, S., Plikus, M.V., and Nie, Q. (2025). CellChat for systematic analysis of cell-cell communication from single-cell transcriptomics. Nat. Protoc. *20*, 180–219. https://doi.org/10.1038/s41596-024-01045-4.
27. Palmieri, L., Ferrand, M., Vu Hong, A., Richard, I., and Albini, S. (2024). In Silico Structural Prediction for the Generation of Novel Performant Midi-Dystrophins Based on Intein-Mediated Dual AAV Approach. Int. J. Mol. Sci. *25*, 10444. https://doi.org/10.3390/ijms251910444.
28. Bourg, N., Vu Hong, A., Lostal, W., Jaber, A., Guerchet, N., Tanniou, G., Bordier, F., Bertil-Froidevaux, E., Georger, C., Daniele, N., et al. (2022). Co-Administration of Simvastatin Does Not Potentiate the Benefit of Gene Therapy in the mdx Mouse Model for Duchenne Muscular Dystrophy. Int. J. Mol. Sci. *23*, 2016. https://doi.org/10.3390/ijms23042016.
29. He, W., and Dai, C. (2015). Key Fibrogenic Signaling. Curr. Pathobiol. Rep. *3*, 183–192. https://doi.org/10.1007/s40139-015-0077-z.
30. Wu, D., Liu, X., and Jin, Z. (2023). Placental mesenchymal stem cells-secreted proenkephalin suppresses the p38 MAPK signaling to block hyperproliferation of keloid fibroblasts. Tissue Cell *85*, 102218. https://doi.org/10.1016/j.tice.2023.102218.
31. Fuchs, M.A.A., Schrankl, J., Wagner, C., Daniel, C., Kurtz, A., and Broeker, K.A.E. (2023). Localization and characterization of proenkephalin-A as a potential biomarker for kidney disease in murine and human kidneys. Biomarkers *28*, 76–86. https://doi.org/10.1080/1354750X.2022.2146196.

32. Villalta, S.A., Nguyen, H.X., Deng, B., Gotoh, T., and Tidball, J.G. (2009). Shifts in macrophage phenotypes and macrophage competition for arginine metabolism affect the severity of muscle pathology in muscular dystrophy. Hum. Mol. Genet. *18*, 482–496. https://doi.org/10.1093/hmg/ddn376.

33. Vidal, B., Serrano, A.L., Tjwa, M., Suelves, M., Ardite, E., De Mori, R., Baeza-Raja, B., Martínez de Lagrán, M., Lafuste, P., Ruiz-Bonilla, V., et al. (2008). Fibrinogen drives dystrophic muscle fibrosis via a TGFbeta/alternative macrophage activation pathway. Genes Dev. *22*, 1747–1752. https://doi.org/10.1101/gad.465908.

34. Wehling-Henricks, M., Jordan, M.C., Gotoh, T., Grody, W.W., Roos, K.P., and Tidball, J.G. (2010). Arginine metabolism by macrophages promotes cardiac and muscle fibrosis in mdx muscular dystrophy. PLoS One *5*, e10763. https://doi.org/10.1371/journal.pone.0010763.

35. Vetrone, S.A., Montecino-Rodriguez, E., Kudryashova, E., Kramerova, I., Hoffman, E.P., Liu, S.D., Miceli, M.C., and Spencer, M.J. (2009). Osteopontin promotes fibrosis in dystrophic mouse muscle by modulating immune cell subsets and intramuscular TGF-beta. J. Clin. Investig. *119*, 1583–1594. https://doi.org/10.1172/JCI37662.

36. Mendell, J.R., Sahenk, Z., Lehman, K.J., Lowes, L.P., Reash, N.F., Iammarino, M.A., Alfano, L.N., Lewis, S., Church, K., Shell, R., et al. (2024). Long-term safety and functional outcomes of delandistrogene moxeparvovec gene therapy in patients with Duchenne muscular dystrophy: A phase 1/2a nonrandomized trial. Muscle Nerve *69*, 93–98. https://doi.org/10.1002/mus.27955.

37. Ertl, H.C.J. (2022). Immunogenicity and toxicity of AAV gene therapy. Front. Immunol. *13*, 975803. https://doi.org/10.3389/fimmu.2022.975803.

38. Corre, G., Seye, A., Frin, S., Ferrand, M., Winkler, K., Luc, C., Dorange, F., Rocca, C. J., and Galy, A. (2022). Lentiviral standards to determine the sensitivity of assays that quantify lentiviral vector copy numbers and genomic insertion sites in cells. Gene Ther. *29*, 536–543. https://doi.org/10.1038/s41434-022-00315-8.

OMTM, Volume *33*

Supplemental information

FAP-CAR-T cells reduce dystrophic muscle fibrosis, improving adeno-associated virus gene transfer efficacy

Maxime Ferrand, Céline J. Rocca, Guillaume Corre, Valentina Buffa, Sophie Frin, Francine Garnache-Ottou, Elodie Bôle-Richard, Sonia Albini, Isabelle Richard, and Anne Galy

Figure S1

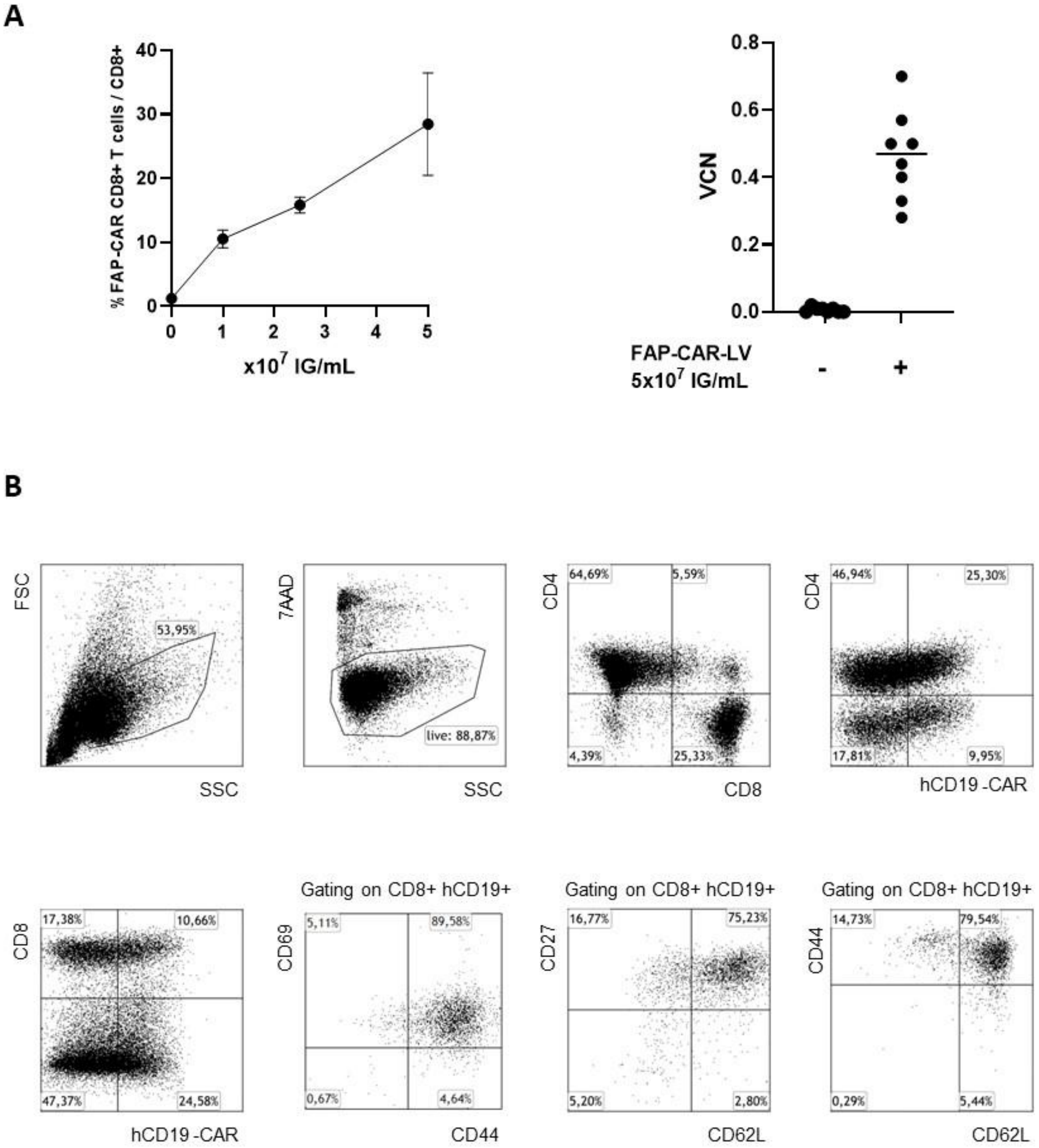

Figure S1 legend: A. Dose effect of the FAP-CAR LV on CD8 T cells transduction. The FAP-CAR LV was tested at 3 concentrations: 1, 2.5 and 5x10^7 IG/mL on 1x10^6 T cells. The left panel shows the percentage of FAP+CD8+ T cells among CD8 T cells measured by flow cytometry after 5 days of transduction, n=3 experiments. The right panel shows the vector copy number per cell (VCN) measured by ddPCR on cells transduced with 5x10^7 IG/mL of FAP-CAR LV, n=8 experiments. **B. Representative dot plots of n=3 different flow cytometry experiments**. Plots show the gating strategy used on spleen CD3+ T cells after 7 days of culture (5 days post transduction). The percentages of live CD8+ and CD4+ cells expressing the CAR with truncated CD19 and among the CD8+ CAR+ cell population, the percentage of activated T cells expressing CD44, CD69, CD27 and CD62L are represented.

Figure S2

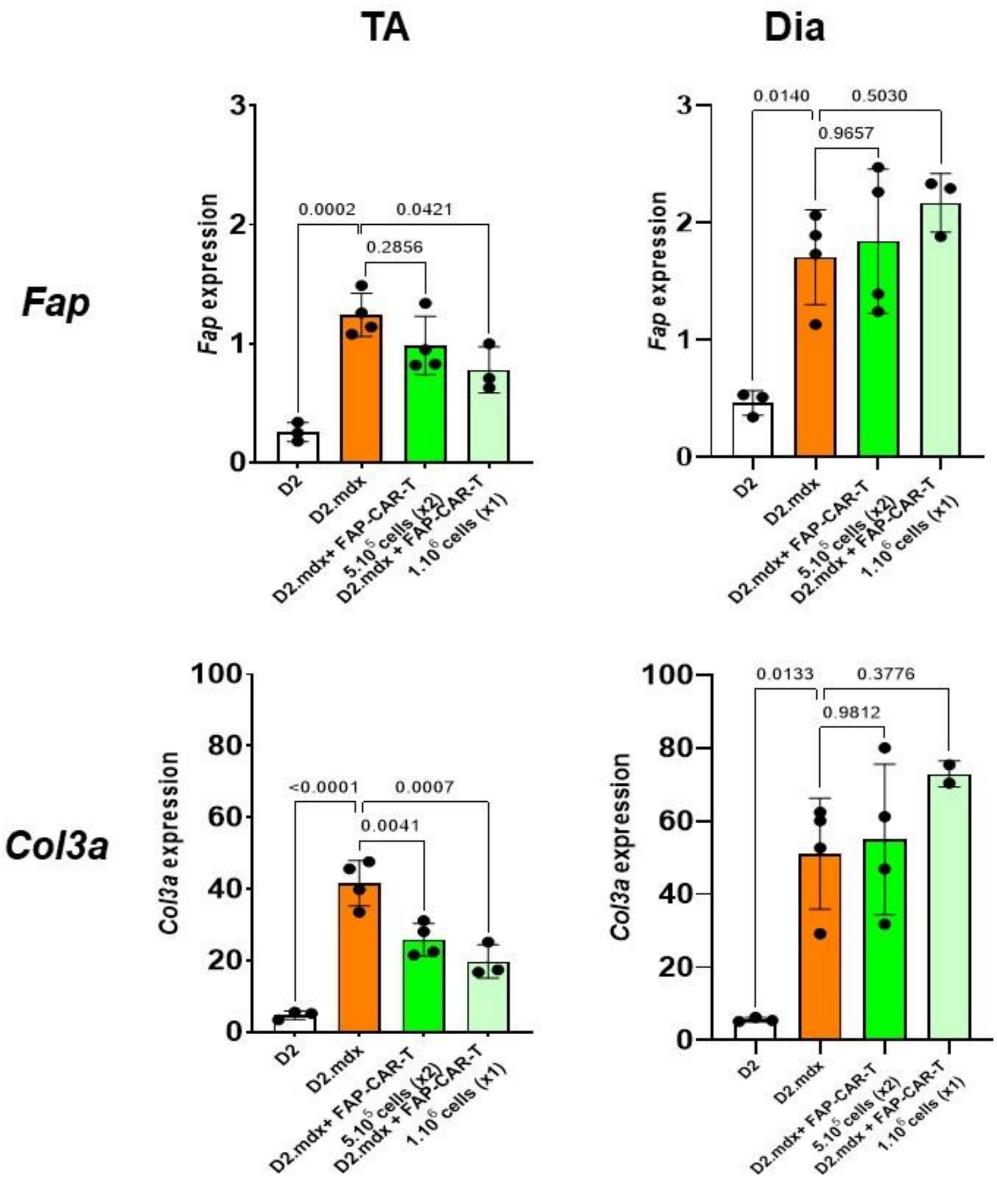

Figure S2 legend: Gene expression after treatment: Graphs show the mRNA expression of the genes *Fap* (top panel) and *Col3a* (bottom panel) in the muscles TA (left panel) or Dia (right panel) of either: D2 mice (white bars) or D2.mdx mice treated with control T cells (orange bars) or D2.mdx mice treated with two doses of $5x10^5$ FAP-CAR-T cells one week apart (dark green bars) or D2.mdx mice treated with one single dose of $1x10^6$ FAP-CAR-T(light green bars). Data represent values from n=3 to 4 mice per group and are relative expressions of the indicated genes normalized to *PO*. Statistical analysis used a one-way ANOVA test.

Figure S3

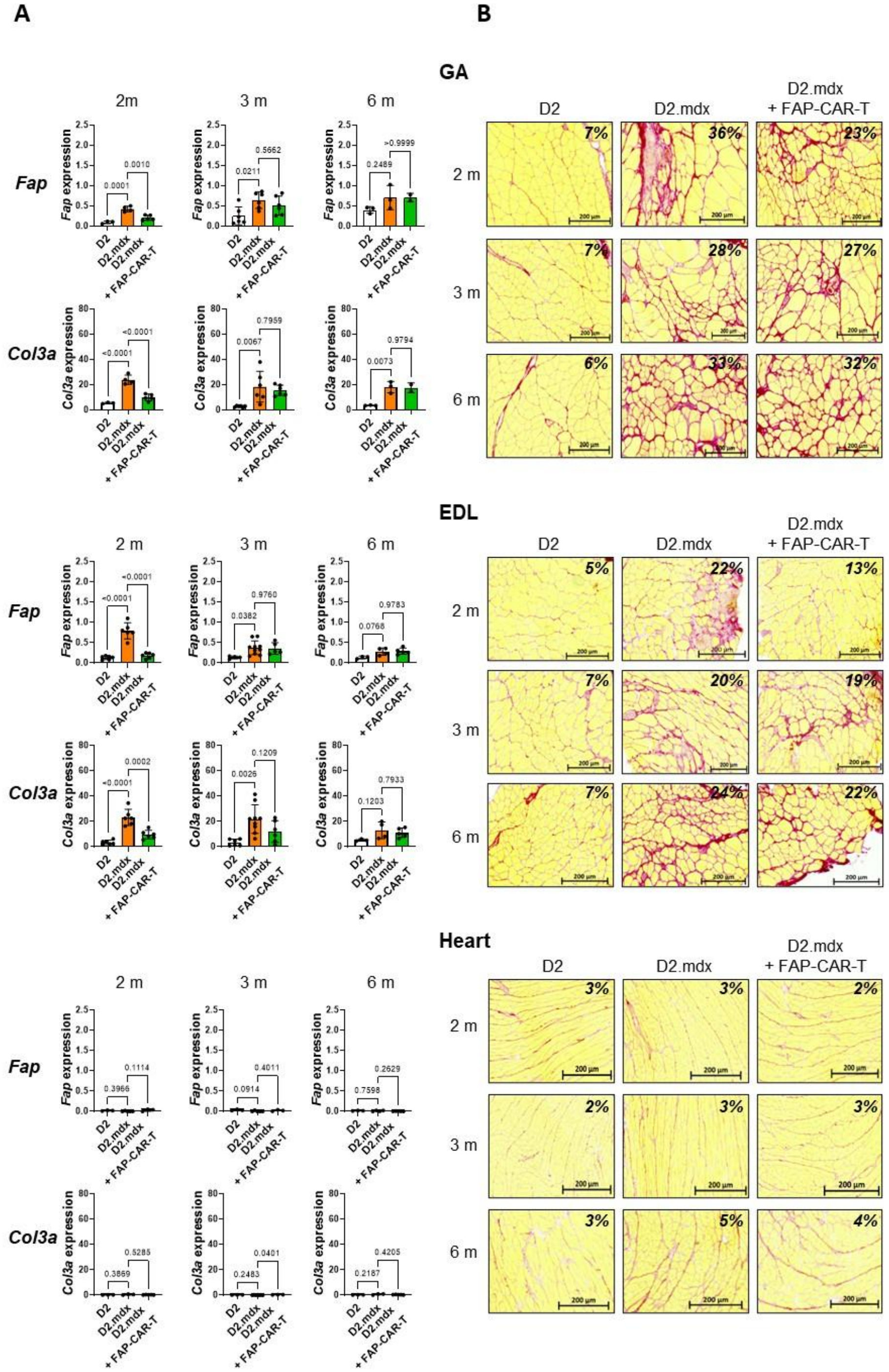

Figure S3 legend: Gene expression (A) and histological findings (B) in different muscles after CAR-T cell treatments in mice of different age. A. mRNA expression of the genes *Fap* (top graphs) and *Col3a* (bottom graphs) in the muscles GA (top panel), *EDL* (middle panel) and heart (bottom panel) from D2 mice (white bars) or D2.mdx mice treated with control T cells (orange bars) or with FAP-CAR-T cells (green bars) at different ages of life (n=3 to 10 mice per group). Values are relative expression normalized to *PO*. Statistical

analysis: One-way ANOVA statistic test. **B.** Representative images of Sirius red histological sections of GA, heart and EDL from mice treated as in (A). Indicated values represents the percentage of area occupied by collagen deposits relative to the total area of muscle section. The red intensity staining reveals collagen deposits (Objective: 10X).

Figure S4

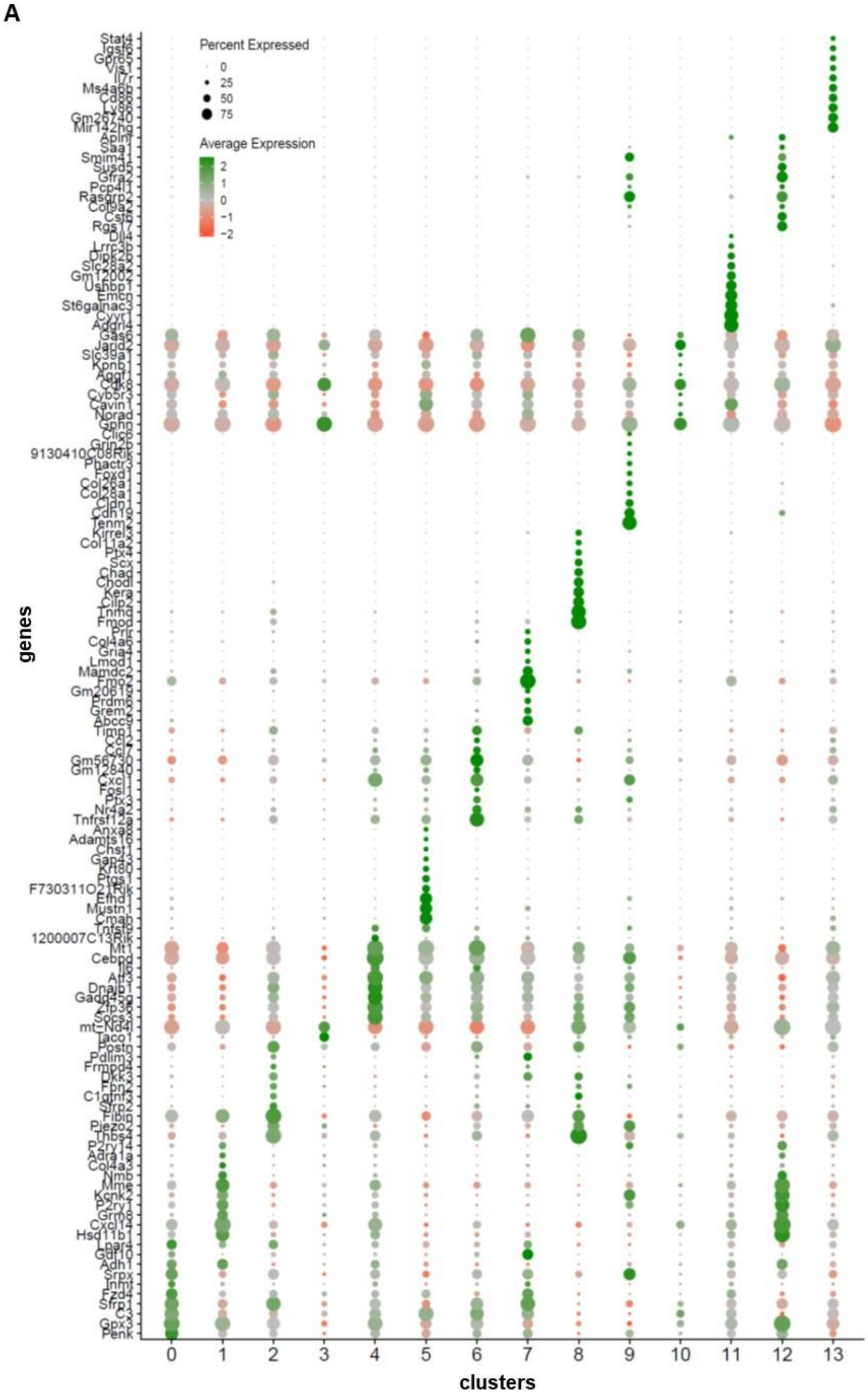

Figure S4 legend: Dot plot Seurat in different categories of cells, based on single cell RNA seq data. The row-scaled expression of top 10 or top 20 expressed genes is shown. The size of each dot represents the percentage of cells within the indicated cluster that express the gene. Larger dots indicate that a higher proportion of cells in the cluster express the gene, while smaller dots indicate a lower proportion. The

color intensity of each dot reflects the average expression level of the gene within the cluster. Greener colors represent higher average expression levels, while reder colors

A. Top 10 expressed genes on *Pdgfra* sub-clusters (i.e clusters 0 to 13).

Figure S4 (continued)

B

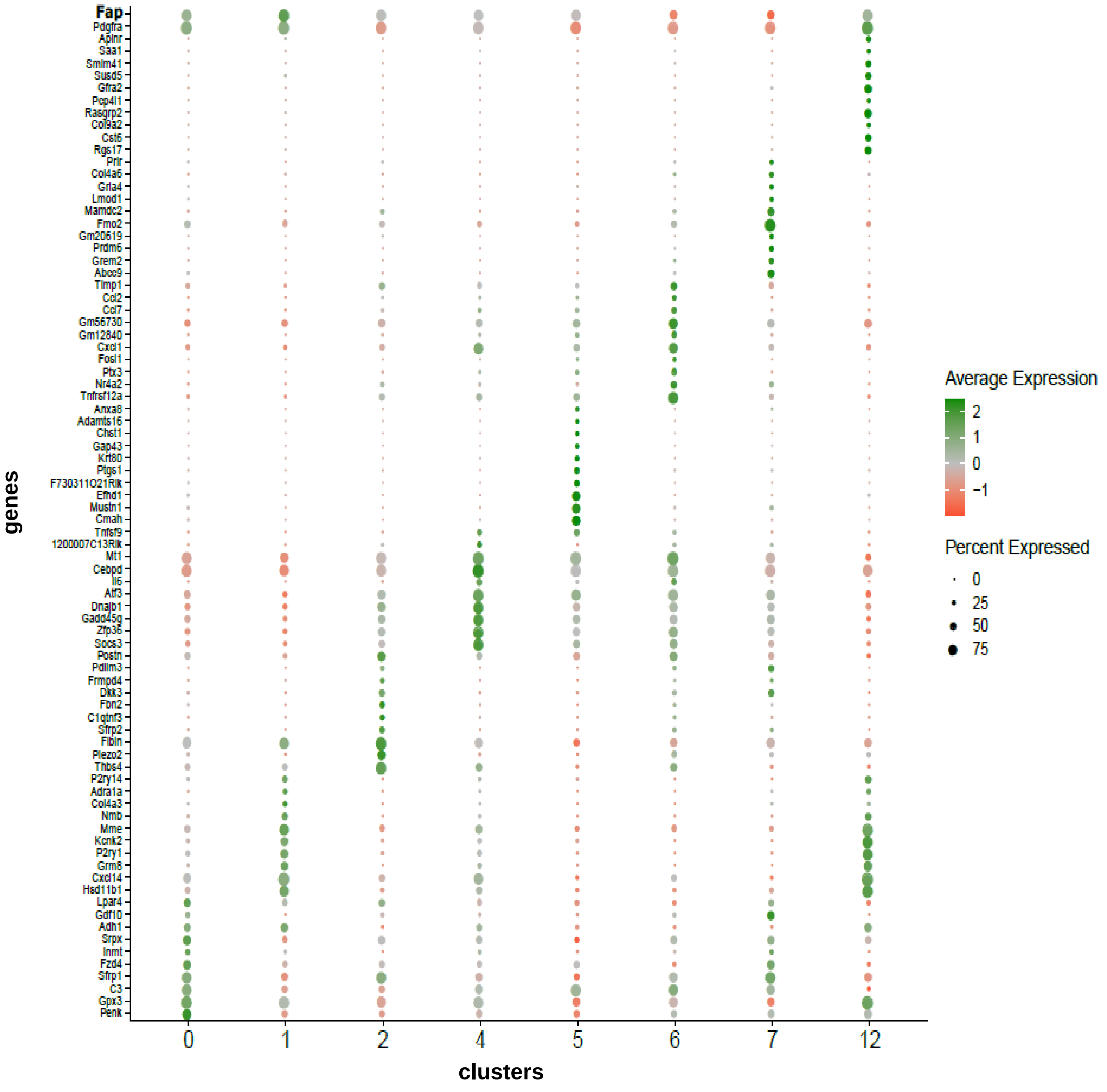

B. Top 10 expressed genes on FAP cells sub-clusters (i.e clusters 0,1,2,4,5,6,7 and 12).

Figure S4 (continued)

C

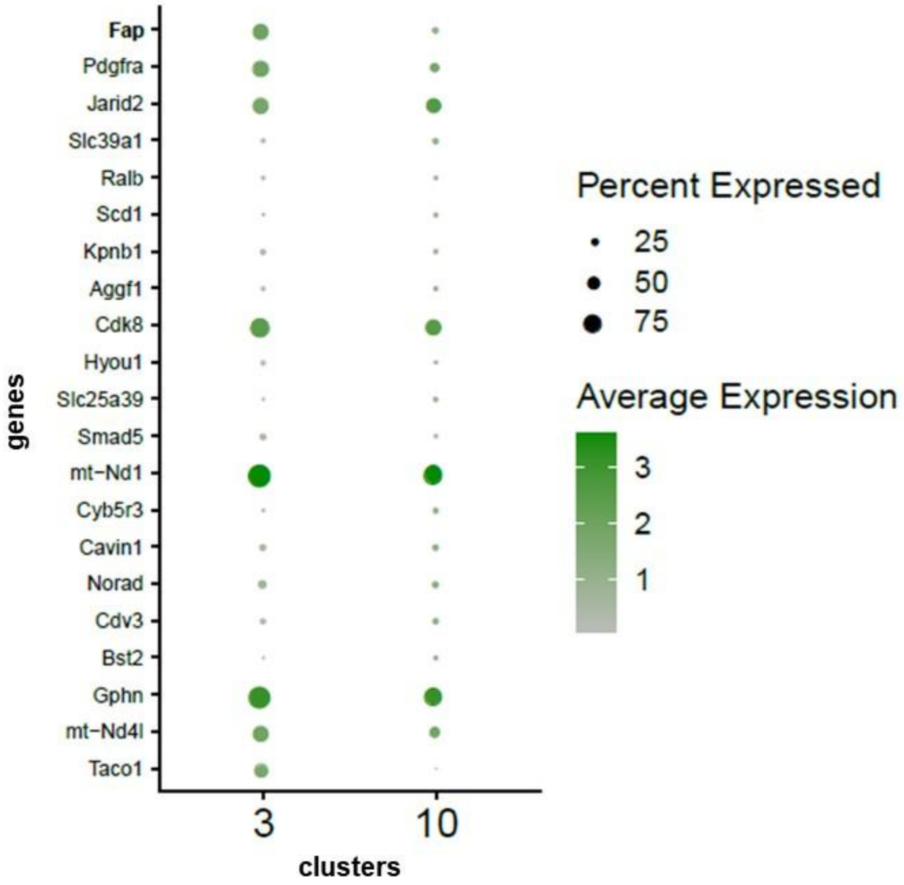

C. Top 20 expressed genes on fibroblasts sub-clusters (i.e clusters 3 and 10).

Figure S4 (continued)

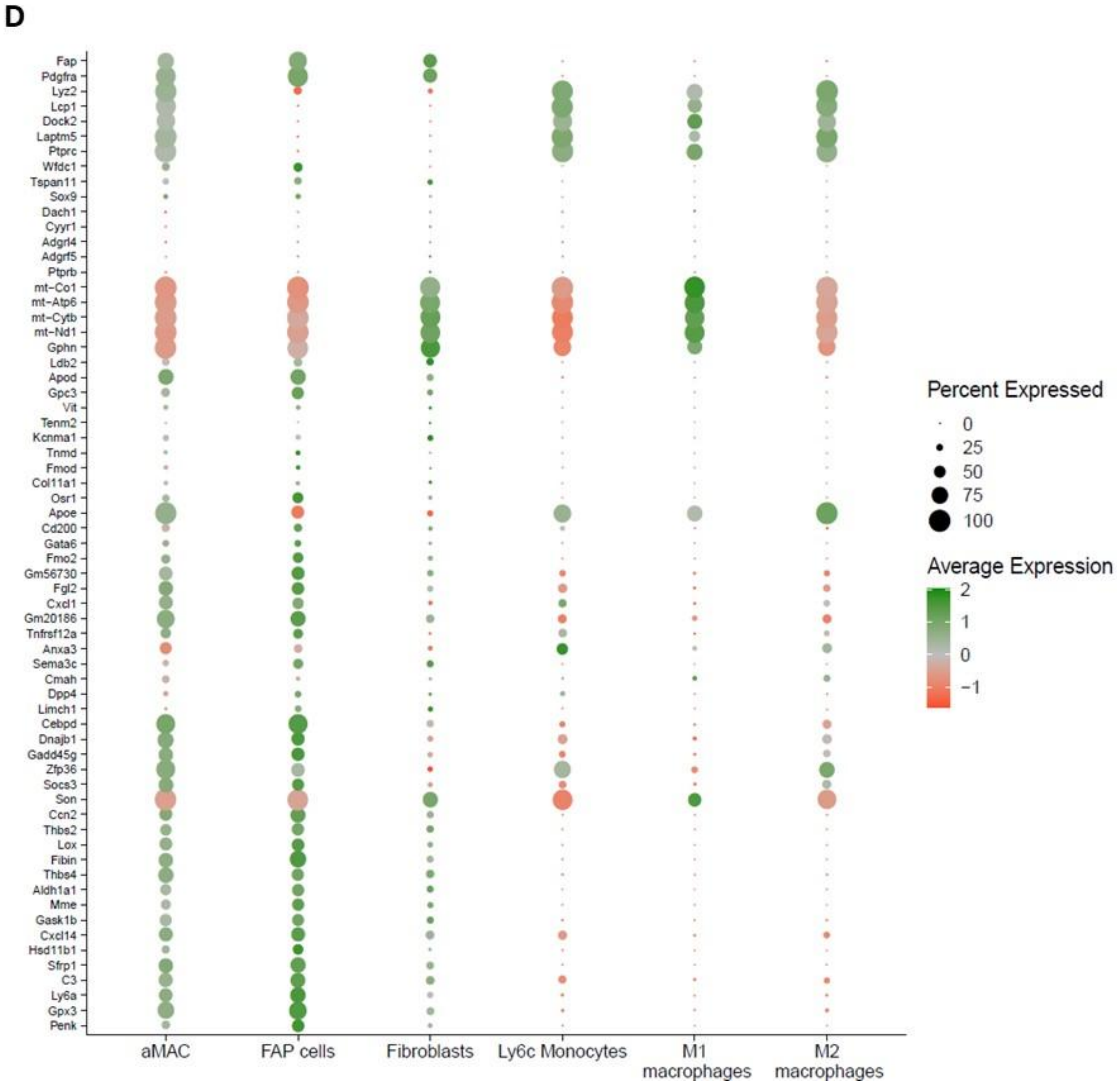

D. Top 20 expressed genes on atypical macrophages (aMAC), FAP cells, Fibroblasts, Ly6c monocytes, M1 macrophages and M2 macrophages clusters.

Figure S5

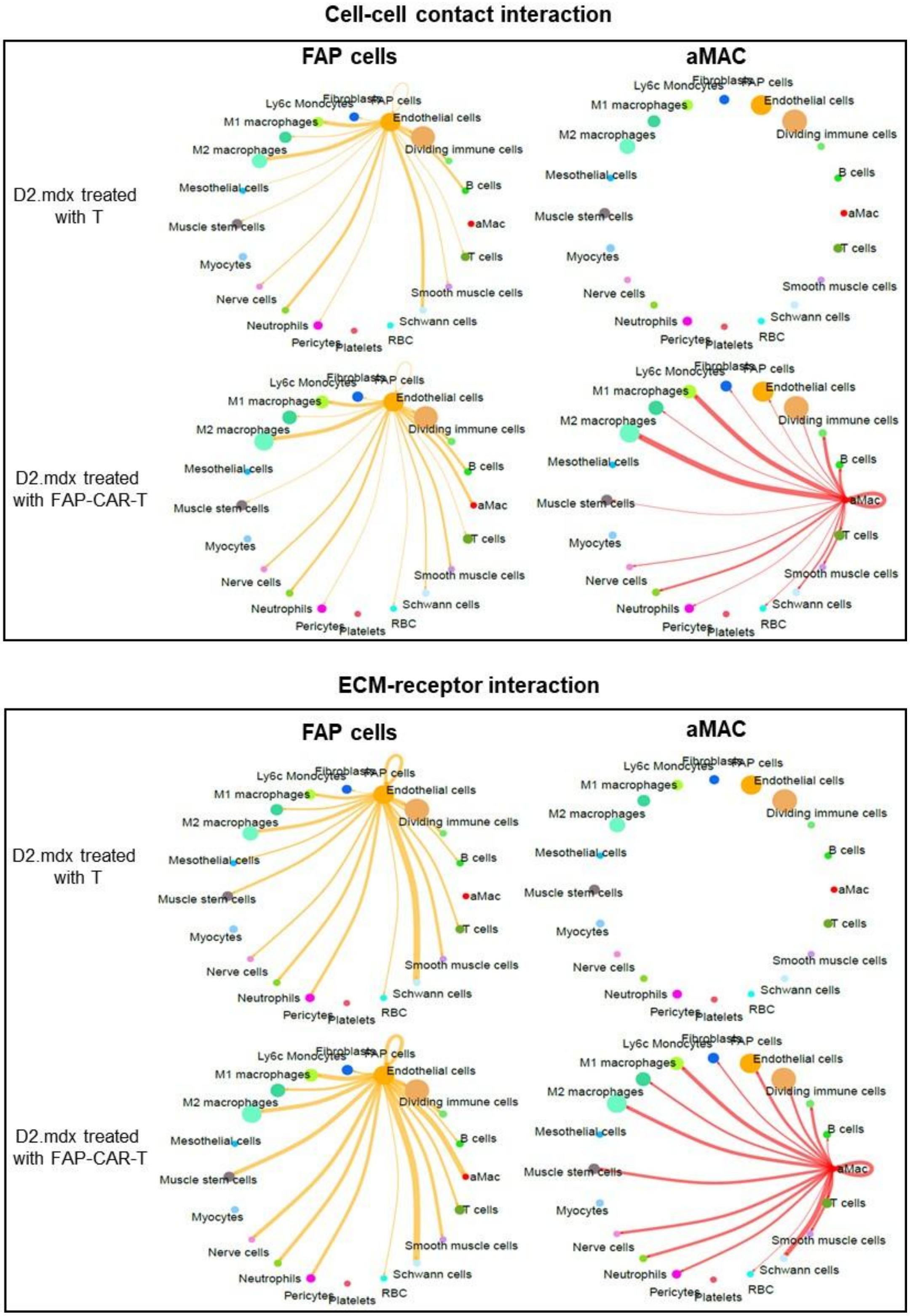

Figure S5 legend: CellChat analysis of cellular interactions as cell-cell contact (top panels) or cell receptor-extracellular matrix (ECM) interactions (bottom panels) from single cell transcriptomic data.

Circle plot showing the intercellular communication strength between major cell types for FAP cells (left panel) and aMAC cells (right panel) in D2.mdx mice treated with control T cells or D2.mdx mice treated with FAP-CAR-T cells. The lines indicate ligand receptor interactions between the different cell types. The thickness of the line is proportional to the probability of interaction between two cell types based on the number, the weight and the strength of ligand-receptor interactions, with loops representing autocrine circuits.

Figure S6

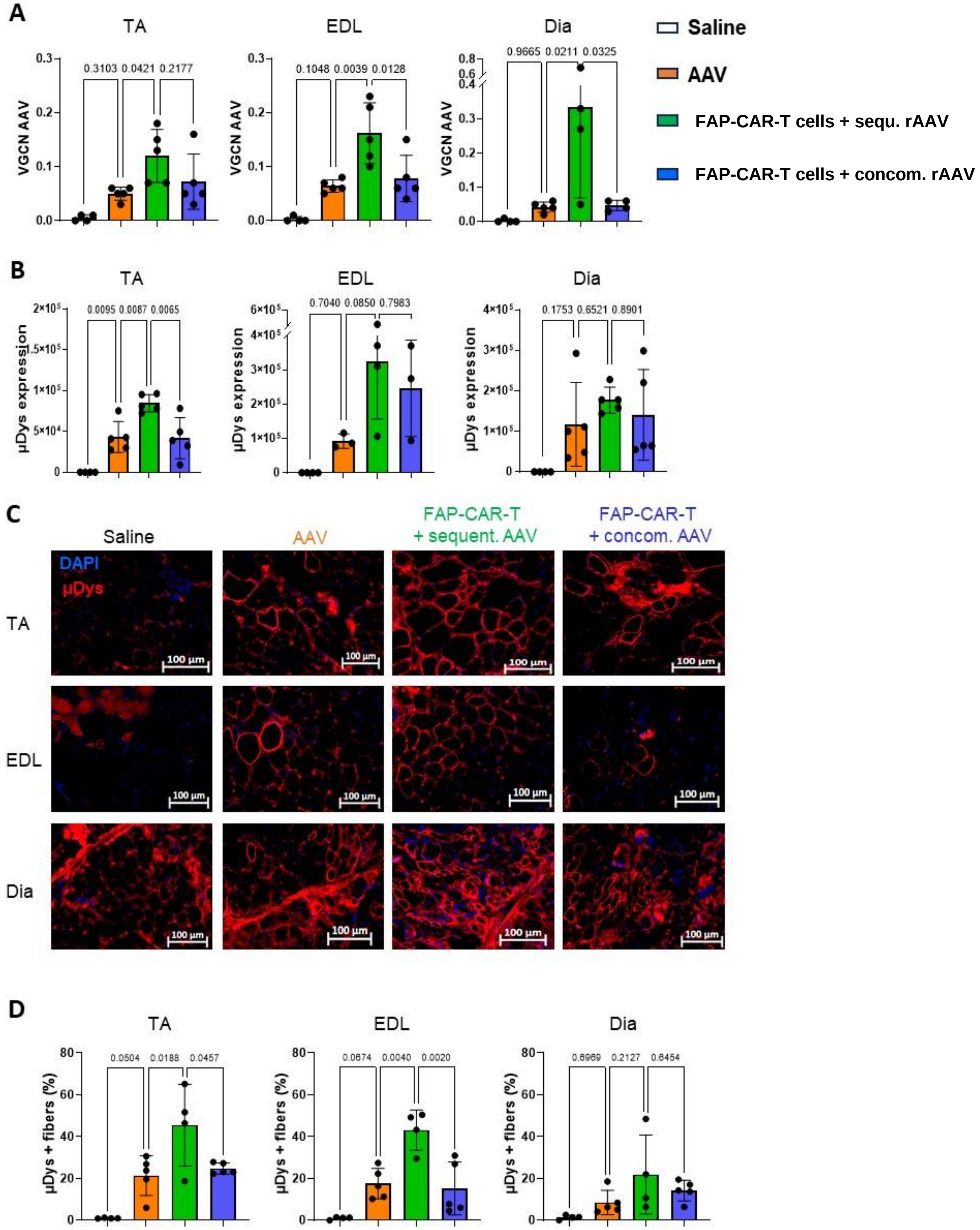

Figure S6 legend: Temporal effects of CAR-T cell administration in combination with gene therapy. D2.mdx mice were injected with two consecutive doses ($1x10^6$) of FAP-CAR-T cells and with 1 dose (5.10^{12}

vg/kg) of rAAV9-microdystrophin vector given sequentially (2 weeks after the last dose of CAR-T cells) or concomitantly (with the second dose of CAR-T cells). Control mice received only rAAV (orange bars) or saline (open bars). **A.** Quantification of vector genome viral copy number (VGCN) with ddPCR in TA, EDL and Dia in D2.mdx mice. **B.** Quantification of microdystrophin (µDys) expression after capillary western with DYS-B antibody in TA, EDL and Dia at 6 weeks post-injection in mice. Values are represented as normalized area under the curve (AUC). **C.** Representative immunohistology of microdystrophin (µDys) in the TA, EDL and Dia at 6 weeks post-injection**. D.** Quantification of dystrophin positive myofibers in TA, EDL and Dia at 6 weeks post-injection in D2.mdx mice. The percentage of dystrophin+ fibers is represented of number of dystrophin positive fibers over fibers positive for laminin. For all the panels: *N* = 3-5 mice. For panel (**A**–**F**): One-way ANOVA statistic test.

Table S1: **Oligonucleotide sequences**

Oligo	Sequence 5' –> 3'
Albumin.fw	GCTGTCATCTCTTGTGGGCTGT
Albumin.rv	ACTCATGGGAGCTGCTGGTTC
Albumin.pr	CGCACGGCAAGAGGCGAGG
Provirus.fw	CACTCCCAACGAAGACAAGA
Provirus.rv	TCTGGTTTCCCTTTCGCTTT
Provirus.pr	TCTCTAGCAGTGGCGCCCGAACAGG
mTitin.fw	AAAACGAGCAGTGACGTGAGC
mTitin.rv	TTCAGTCATGCTGCTAGCGC
mTitin.pr	TGCACGGAAGCGTCTCGTCTCAGTC
ITRG, AAV22mers.fw	CTCCATCACTAGGGGTTCCTTG
ITRG, AAV18mers.rv	GTAGATAAGTAGCATGGC
ITRG, AAV_MGB.pr	TAGTTAATGATTAACCC
Microdystrophin.fw	GGTTGTGCTGGTCCAGGGCGT
Microdystrophin.rv	CCAACAAAGTGCCCTACTACATC
Microdystrophin.pr	CCGAGCTGTACCAGAGCCTGGCC
mPO MH181P0.fw	CTCCAAGCAGATGCAGCAGA
mPO M267PO.rv	ATAGCCTTGCGCATCATGGT
mPO M225PO.pr	CCGTGGTGCTGATGGGCAAGAA
MPZ (PO) ddPCR Gene Expression Assay	ID: dMmuCPE5103243, Biorad
FAP ddPCR Gene Expression Assay	ID: dMmuCPE5093936, Biorad
Col3a ddPCR Gene Expression Assay	ID: dMmuCPE5110694, Biorad

Table S2: **List of antibodies used**

Specificity	Clone / Reference	Conjugate	Provider	Dilution
mouse CD3	17A2	Fitc	BD Pharmingen	1 :100
mouse CD4	RM4.5	Pacific blue	BD Pharmingen	1 :200
mouse CD8a	53-6.7	PE-Cy7	Invitrogen	1 :200
mouse CD69	H1.2F3	Fitc	BD Pharmingen	1 :150
mouse CD44	IM7	PE	BD Pharmingen	1 :100
mouse CD62-L	MEL-14	APC	BD Pharmingen	1 :100
human CD19	HIB19	Alexa 700	BD Pharmingen	1 :10
human CD19	J3-119	APC	Beckman Coulter	1 :10
mouse CD27	LG.3A10	BV510	BD Horizon	1 :100
mouse CD107a	1D4B	PE	BD Pharmingen	1 :20
mouse laminin	polyclonal	unconjugated	Dako	1 :1000
human dystrophin	34C5	unconjugated	Leica Biosystem	1 :100
mouse CD11b	M1/70	unconjugated	BD Pharmingen	1 :40
mouse FAP	polyclonal	unconjugated	Invitrogen	1 :200
goat anti-rat	polyclonal secondary	Alexa Fluor 488	Invitrogen	1 :600
donkey anti-mouse	polyclonal secondary	Alexa Fluor 594	Invitrogen	1 :1000
